# Manifestations of systemic disease in the retina and fundus of cats and dogs

**DOI:** 10.3389/fvets.2024.1337062

**Published:** 2024-02-20

**Authors:** Billie Beckwith-Cohen, Simon M. Petersen-Jones

**Affiliations:** Department of Small Animal Clinical Sciences, College of Veterinary Medicine, Michigan State University, East Lansing, MI, United States

**Keywords:** chorioretinitis, retinitis, retinal detachment, dog, cat, ophthalmology, retinopathy

## Abstract

The fundus is unique in that it is the only part of the body that allows for a noninvasive and uninterrupted view of vasculature and nervous tissue. Utilization of this can be a powerful tool in uncovering salient incidental findings which point to underlying systemic diseases, and for monitoring response to therapy. Retinal venules and arterioles allow the clinician to assess changes in vascular color, diameter, outline, and tortuosity. The retina and optic nerve may exhibit changes associated with increased or decreased thickness, inflammatory infiltrates, hemorrhages, and detachments. While some retinal manifestations of systemic disease may be nonspecific, others are pathognomonic, and may be the presenting sign for a systemic illness. The examination of the fundus is an essential part of the comprehensive physical examination. Systemic diseases which may present with retinal abnormalities include a variety of disease classifications, as represented by the DAMNIT-V acronym, for Degenerative/Developmental, Anomalous, Metabolic, Neoplastic, Nutritional, Inflammatory (Infectious/Immune-mediated/ischemic), Toxic, Traumatic and Vascular. This review details systemic illnesses or syndromes that have been reported to manifest in the fundus of companion animals and discusses key aspects in differentiating their underlying cause. Normal variations in retinal anatomy and morphology are also considered.

## Introduction

The examination of the fundus may be brief, or extensive. It is achievable in a darkened room using a variety of instruments including a Finoff transilluminator or indirect ophthalmoscope with a condensing lens, a direct or panoptic ophthalmoscope, a specialized retinal camera, or nowadays a smartphone (1). A thorough assessment is made possible when the pupil is dilated and the optical media before the fundus is fairly clear. Therefore, systemic diseases which manifest in profound uveitis or complete cataracts may hinder the ability to appreciate changes which occur in the fundus, though some retinal changes may be appreciated indirectly via ultrasonographic techniques, other imaging modalities or histopathology. In addition, contraindications to using mydriatics (e.g., glaucoma, lens luxation), challenges with pupillary dilation (e.g., pharmacologic or pathologic miosis) may hinder the exam. It is of note, that at times even a partial view of the fundus can offer valuable insight.

**Figure 1:**
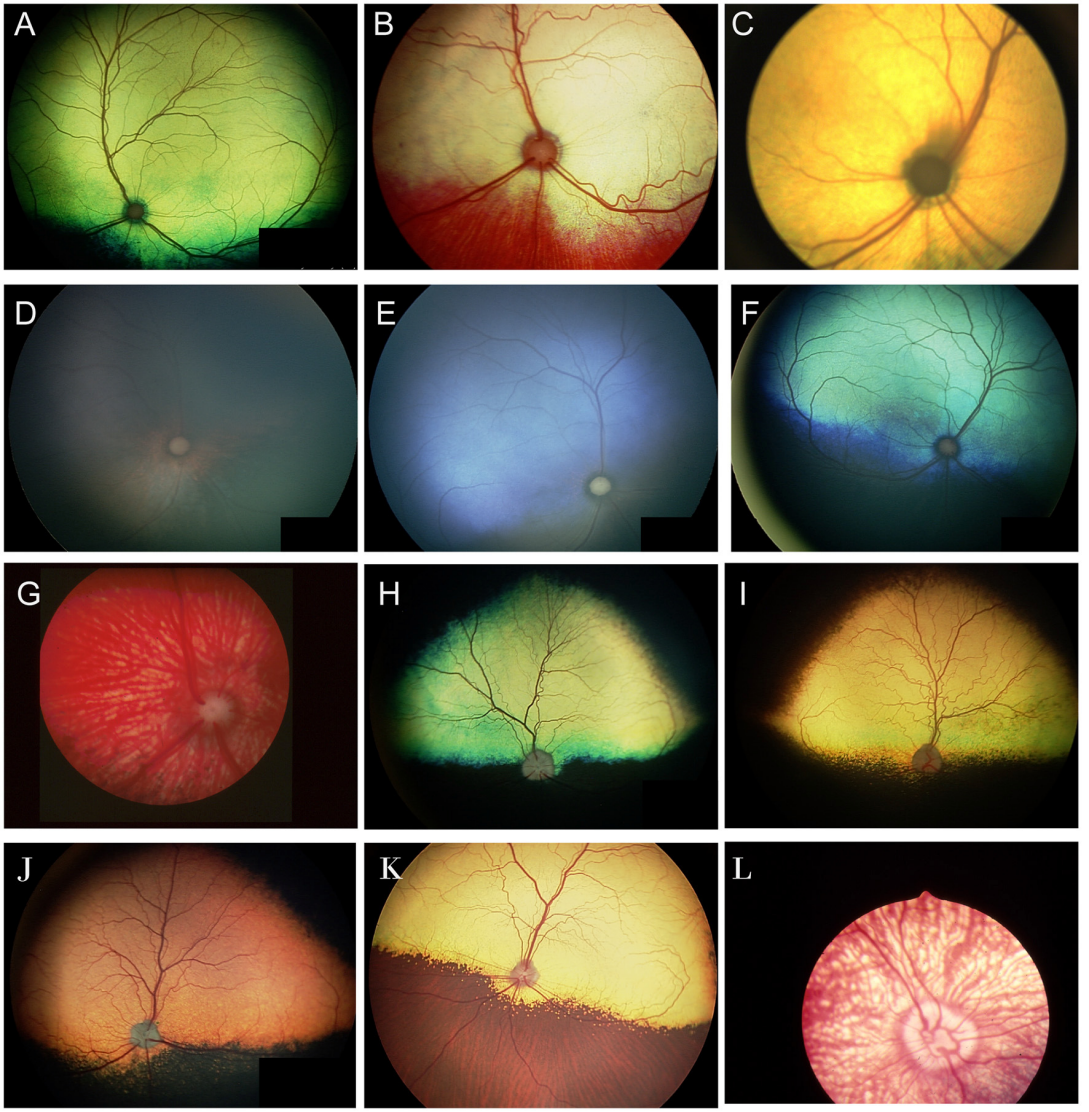
The normal fundus of the cat **(A–G)** and dog **(H–L)**. **(A)** Normal fundus of a cat with a green-yellow tapetum. The feline tapetum is large and spans the majority of the dorsal retina extending beyond the optic nerve ventrally. **(B)** A feline with a subalbinotic fundus manifesting with a yellow tapetum and lacking clinically noticeable pigment in the retinal pigment epithelium (RPE) ventrally. Without pigment and tapetum choroidal vasculature is exposed in the ventral retina. **(C)** A closer view of the optic nerve head of a ginger-colored cat exhibits flame shaped gray radiations from the optic nerve head following the tracks of the nerve fiber layer. These represent excessive myelination and are considered clinically insignificant. **(D–F)** Maturation of the feline fundus at 3 weeks of age **(D)**, 6 weeks of age **(E)** and 3 months of age **(F)**. With maturation the tapetum becomes thicker and more refractile and changes in color from a bluish gray to the final color which is most often green or yellow. The myelination of the optic disc also increases. Similar changes are seen in the dog. **(G)** Subalbinotic fundus of a cat with an absent tapetum and scant pigmentation in the RPE ventrally. Note how normal feline retinal vasculature emanates from the margins of the optic nerve head. The optic pit is seen as a focal gray dot in the center of the optic nerve head. The straight streaks typical of the choroidal vasculature have lent it the term tigroid fundus. **(H)** Heavily pigmented fundus with a green-yellow tapetum from a Beagle. **(I)** Heavily pigmented fundus of a dog with a yellow tapetum. **(J)** Heavily pigmented fundus with an amber colored tapetum from an Old English sheepdog. **(K)** Subalbinotic canine fundus with a yellow tapetum and reduced pigment in the RPE of the nontapetal fundus exposing the tigroid appearance of the choroidal vasculature. **(L)** A closer view of the well-myelinated optic disc of a subalbinotic canine fundus with no tapetum and scant pigment in the RPE. Note how the retinal vasculature traverses the optic nerve head and courses around the optic pit.

When examining the fundus, one must be familiar with normal variations in anatomy for the species being examined, and normal breed-related phenotypic variations within certain species. Variations are mostly associated with the appearance and presence of vasculature, tapetum, pigment in the retinal pigment epithelium, and optic nerve head myelination (Figure 1). Unlike humans, the cat and dog do not have a macula or fovea, however they do have a visual streak, and a region analogous to the macula which is called the area centralis. The area centralis is located temporal and dorsal to the optic nerve in dogs and cats and is rich in cone photoreceptors making it important for high acuity vision, and subject to higher metabolic needs (2). The extent of normal retinal vasculature of cats and dogs span the entire retina (i.e., they have a holangiotic retina). The tapetum may be absent, or vary in size, shape, or color, and variations are related to species, breed, and the degree of ocular pigmentation (Figure 1). Poorly pigmented or subalbinotic individuals or eyes tend to have a smaller, patchy, or even absent tapetum (Figures 1G, L). Tapetal changes are also noted with age as the tapetum develops and matures with resulting color change over the first few months of life (Figures 1D–F). The optic nerve head may vary in shape, color, size, depth, and degree of myelination. In dogs the optic nerve is more heavily myelinated than in cats, it has a more triangular shape, as opposed to the round optic nerve head in cats, and is traversed by vessels. Myelination is also age dependent and increases in the first months during maturation. Given the extent of normal variability one must become adept in routine examinations of the fundus to diagnose abnormalities when present. Furthermore, subtle changes may become evident only when comparing the contralateral eye, though this can be challenging in animals with heterochromatic eyes, which is seen more commonly in certain breeds such as the Husky and Collie dogs. In heterochromia, blue eyes have a more pigment dilute fundus and may have a smaller or absent tapetum, while the brown eyes may be heavily pigmented, or variably subalbinotic and poorly pigmented.

The purpose of this review is to familiarize the reader with specific and nonspecific retinal findings associated with naturally occurring systemic diseases, and key aspects which may facilitate arriving at a differential diagnosis using tools which are available at most practices. While retinal manifestations of disease occur in a broad range of companion animals the review will focus on those that occur in cats and dogs. Retinal abnormalities will be classified using the DAMNIT-V acronym (Table 1), with some findings being consistent with more than one classification. While we include a comprehensive review of the literature, it is not exhaustive. Exceedingly rare manifestations, equivocal disease associations and experimentally induced retinal abnormalities are beyond the scope of this review.

**Table 1:** Retinal abnormalities in dogs and cats classified using the DAMNIT acronym.

**Acronym**	**Classification**	**Disease/disorder**	Affected species^*^
D	Degenerative	*Progressive retinal atrophy*	D, C
		*Lysosomal Storage diseases*	D, C
	Developmental	*Oculoskeletal dysplasia*	D
A	Anomalous	*Retinal dysplasia*	D, C
		*Merle ocular dysgenesis*	D
M	Metabolic	*Lipemia retinalis*	D, C
		*Diabetic retinopathy*	D, C
		*Hypertensive retinopathy*	D, C
N	Nutritional	*Taurine deficiency*	C
		*Vitamin A deficiency*	D
		*Vitamin E deficiency*	D
		*Lipemia retinalis*	D, C
	Neoplastic	*Metastatic: Lymphosarcoma*,	D, C
I	Infectious	*Systemic mycoses: Blastomycosis, Histoplasmosis, Cryptococcus* *Tick-borne: Ehrlichiosis, Anaplasmosis, Borreliosis, Babesiosis, Bartonellosis. Encephalitozoonosis*. *Viral: Canine distemper, Canine adenovirus, CHV* *FIP, FeLV, FIV* *Protozoal: Leishmaniasis, Toxoplasmosis and Neosporosis* *Parasitic: Larval migrans* *Algal disease: Prototheca*	D, C
	Immune-mediated	*Uveodermatologic syndrome, Autoimmune retinopathy, SARDS*	D
T	Traumatic	*Ischemic retinopathy*	D, C
	Toxic	*Ivermectin toxicity*	D, C
		*Fluoroquinolone toxicity*	C
V	Vascular	*Coagulopathy*	D, C
		*Thrombocytopenia*	D
		*Anemic retinopathy*	C
		*Hypertensive retinopathy*	D, C
		*Hyperviscosity*	D, C

^*^D, dog; C, cat.

## Degenerative

### Progressive retinal atrophy

**Figure 2:**
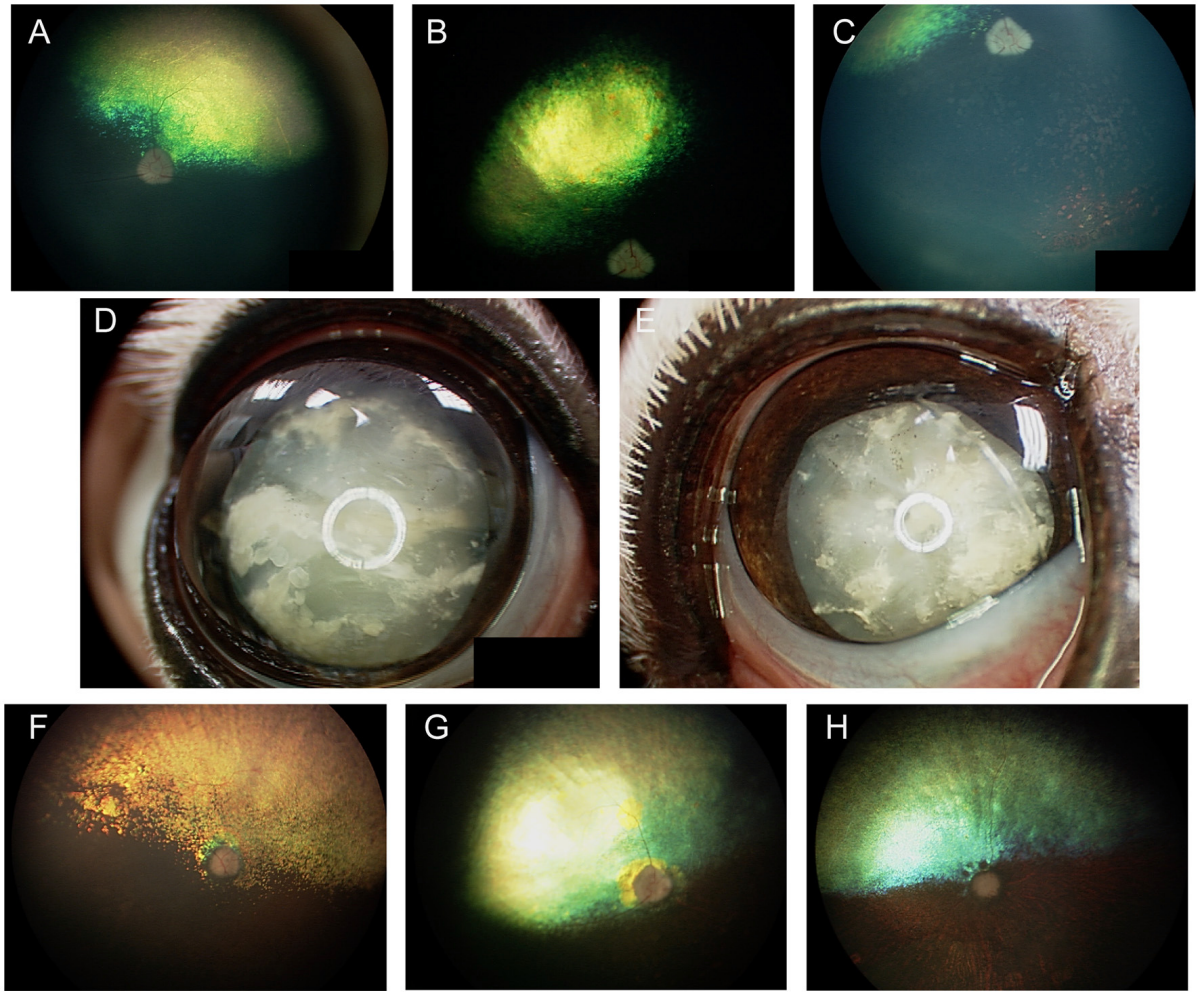
Progressive retinal atrophy in the dog. **(A)** Retinal atrophy in a 1-year-old Beagle-Corgi research dog homozygous for a mutation in *PDE6A*. The tapetum is hyperreflective from loss of retinal thickness and the vasculature is attenuated. **(B, C)** retinal images from the same dog seen in **(A)** at 2.5 years of age. Retinal atrophy, tapetal hyperreflectivity and vascular attenuation have progressed. **(C)** The ventral fundus shows pigmentary changes including multifocal punctate pigment loss. **(D)** Right and **(E)** left eyes of the same dog as seen in **(A–C)** at 10 years of age. There is a secondary hypermature cataract in both eyes that precludes view of the fundus. This dog had negative dazzle and PLR along with a flat electroretinogram. **(F–H)** End stage PRA seen in dogs during routine clinical examination. **(F)** Retinal thinning, vascular attenuation and optic nerve atrophy is seen. The tinctorial halo seen around the optic nerve head which is also noted in **(G)** is often referred to as a peripapillary conus and can also be seen independent of PRA. **(H)** Profound retinal thinning, vascular attenuation and optic nerve atrophy from PRA.

Progressive retinal atrophy is a term which refers to a group of inherited retinal degenerations which worsen or progress over time. They result in bilateral generalized retinal thinning manifest as tapetal hyperreflectivity and accompanied by superficial retinal vascular attenuation and eventually optic nerve head atrophy. The majority of the progressive retinal atrophies (PRAs) in dogs represent diseases that are restricted to retinal dysfunction without systemic manifestations (i.e. non syndromic) (3). In people, certain retinal degenerations that affect the cilium in photoreceptors may also result in deafness, infertility, renal dysfunction and other manifestations due to the presence of cilia in other organs (4). Ciliopathies have been infrequently associated with syndromic retinal degenerations in dogs and cats, and other organ involvement is seldom reported (3). Syndromic retinal degeneration is reported in dogs affected by mutations in Bardet-Biedl syndrome 2 and 4 genes (*BBS2, BBS4*) and Bardet-Biedl syndrome gene *TTC8* (Tetratricopeptide Repeat Domain 8). The deletion in *TTC8* is reported to cause PRA with accumulation of lipofuscin in golden retriever dogs (5). A close inspection of affected dogs showed that they suffered from clinical and morphological signs similar to those seen in human BBS patients such as obesity, renal abnormalities, sperm defects and anosmia (5). A missense variant in *BBS2* is reported to cause syndromic PRA in Shetland sheepdogs (6). Systemic signs in the affected dogs included uncharacteristic wavy haircoat, upturned nose, dental defects, renal changes and occasional obesity (6). A mutation in *BBS4* was also described in the Hungarian puli resulting in PRA and severe reduction in sperm motility (7). The distribution of retinal atrophy and the rate of degeneration vary greatly by condition, though bilateral similarity is expected (Figure 2). Dogs with advanced long-standing PRA will often develop a cataract which may preclude the ability to see the fundus (Figures 2D, E); the lack of a dazzle response or a flat ERG can facilitate confirmation of co-existing retinal degeneration. While gene augmentation therapy has been developed in canine and feline models of specific human retinal disease, it is not commercially available for veterinary patients (8, 9). Therefore, there is no current treatment for these conditions. While some advocate the use of nutraceuticals and presumed neuroprotective therapies the practice is not supported by strong research evidence (10). Furthermore, as demonstrated in people, supplementation with certain vitamins may have adverse effects on specific retinal diseases and therefore caution is advised when advocating for oral supplements (11). Commercial genetic tests are available for most known forms of PRA in dogs and cats and may help inform the client on the expected course of disease as well as offer a definitive diagnosis. Moreover, genetic testing is a valuable tool for breeders, and it can aid in the reduction or elimination of affected progeny (12). Currently, there is no regulatory oversight for canine clinical genetic testing, though guidelines and standards have been proposed (12).

### Lysosomal storage diseases

A variety of lysosomal storage diseases have been reported both in the dog and cat and are generally divided into mucopolysaccharidoses (MPS), oligosaccharidoses and lipidoses. These conditions are extremely rare in a clinical setting. The substrates of the deficient enzyme accumulate in tissues causing progressive organ damage. The cornea, retina, and lens are most affected in the eye; and the liver, central nervous system, skeletal and smooth muscle including the heart are commonly affected elsewhere in the body, though involvement in other organs is possible. Changes in the fundus may vary according to the conditions which may include mottling, pigmentary accumulation or changes, and retinal atrophy. There is no practical treatment for those rare conditions and interest in them has been largely driven by search for animal models for their human parallels and development of gene therapy (13, 14).

Conditions affecting the retina or fundus in cats include mucolipidosis II (15), GM1-gangliosidosis (16), GM2-gangliosidosis (17), sphingomyelinosis (Niemann-Pick) types A (18) and C (19), α-mannosidosis (20), MPS I (21), MPS VI (22), and neuronal ceroid lipofuscinosis (23).

Conditions affecting the retina or fundus in dogs include neuronal ceroid lipofuscinosis (14, 24), α -L-fucosidosis (25, 26), MPS I (27), MPS IIIA (28), MPS IIIB (29), MPS VI (30), MPS VII (31), GM1-gangliosidosis (32), and GM2-gangliosidosis (33). While MPS II has been reported to be associated with posterior segment abnormalities in people (34), ocular findings were not described in a single published case report of the condition in a dog (35). Similarly, ocular finding were reported in people with glycogenosis II (36) but ocular findings were not described in reported cases in the dog (37). Retinal manifestations are seldom reported in people affected by glucocerebrosidosis (i.e., Gaucher's disease) (38) and were not reported in the single published well-characterized case in a dog (39). Galactosylceramide lipidosis (i.e., globoid cell leukodystrophy, Krabbe disease) has been associated with optic nerve and retinal disease in affected people (40). Affected dogs appear to have a normal appearing fundus though some exhibit postretinal blindness due to occipital lobe and optic radiation inflammatory lesions (41). Ocular findings were not described in 2 case reports of dogs affected by sphingomyelinosis (Niemann-Pick) types A (42) and C (43). Investigation of these conditions may be pursued through genetic testing, or via laboratories which perform metabolic testing.

## Developmental

### Oculoskeletal dysplasia

**Figure 3:**
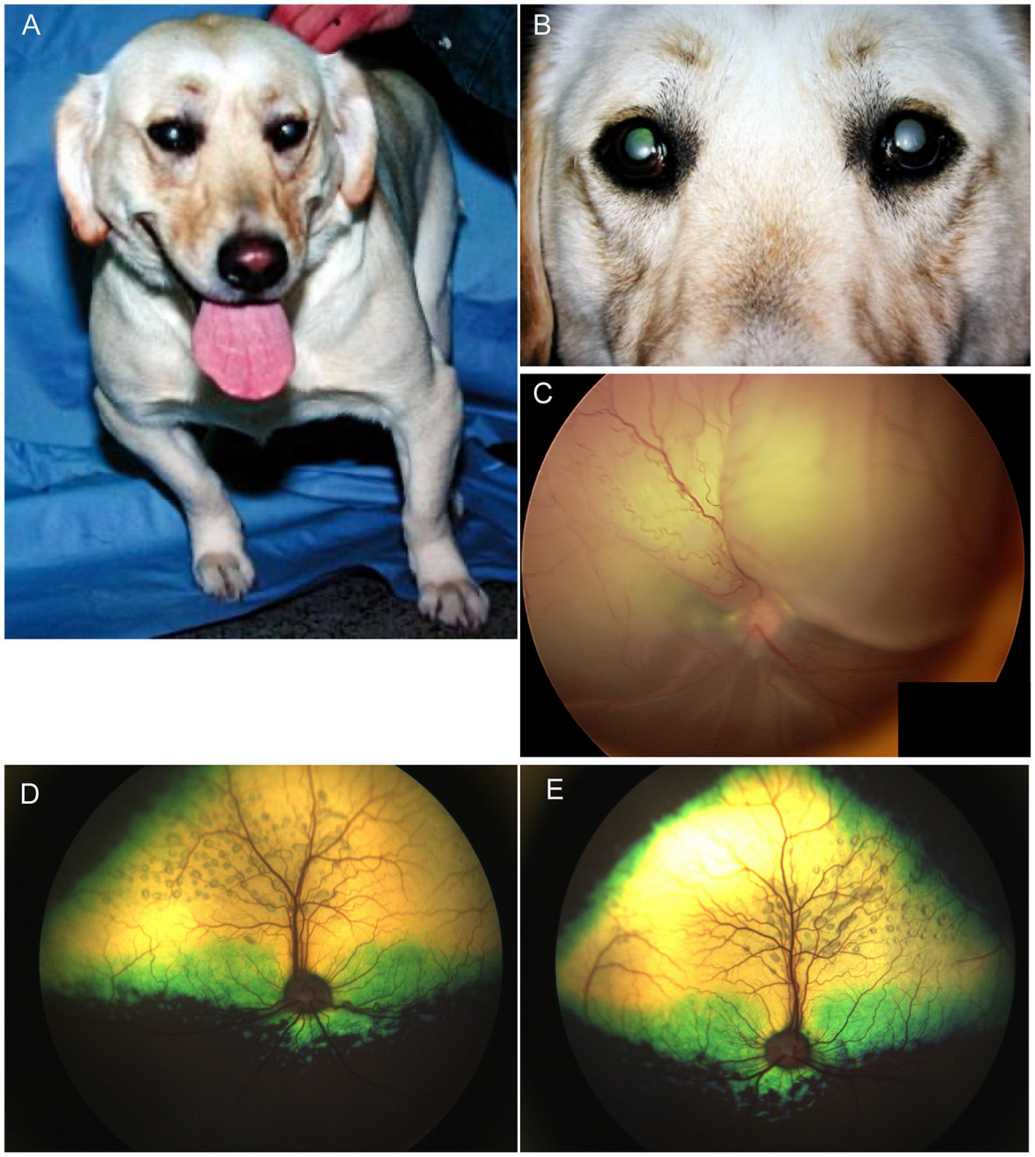
Oculoskeletal dysplasia. **(A)** a Labrador retriever manifesting abnormalities in the appendicular skeleton including short limbs. **(B)** close up image of the dog seen in **(A)** revealed opacities to the lenses in both eyes consistent with immature cataracts. **(C)** Subtotal bollus retinal detachment in the right eye of a 6-year-old Labrador retriever. **(D)** Right and **(E)** left eyes of a Labrador retriever illustrating multifocal retinal rosettes and folds that clustered in the superior retina near the dorsal vasculature. These multifocal retinal dysplasia lesions are typical in dogs heterozygous for mutations that cause oculoskeletal dysplasia.

In Labrador Retrievers and Samoyeds, retinal dysplasia and other ocular abnormalities have been associated with orthopedic abnormalities as part of an inherited oculoskeletal dystrophy, also known as *dwarfism with retinal dysplasia (drd)* (44, 45). The condition has been associated with mutations in the *COL9A2* and *COL9A3* genes for which genetic testing is available, and severity is variable in affected dogs (44). Dogs homozygous for the mutations manifest with osteochondrodysplasia of the appendicular skeleton, short-limbed dwarfism as well as elbow and hip dysplasia (Figure 3A) (44, 45). Ocular lesions include cataracts, retinal degeneration and dysplasia, retinal detachment, vitreous syneresis and persistent hyperplastic primary vitreous (Figures 3B, C) (44, 45). Changes in heterozygous dogs are usually limited to the retina and vitreous, and retinal imaging from Labrador retrievers heterozygous for *drd1* or *drd2* mutations showed multifocal retinal folds that clustered in the superior retina near the dorsal vasculature (Figures 3D, E) (46). By contrast, short stature without radiological evidence of dwarfism, and concurrent retinal dysplasia has been reported in American pit bull terriers for which an autosomal dominant mode of inheritance was suggested (47). While the causative gene was not identified, mutations in *COL9A2* and *COL9A3* were ruled out (47). Ocular changes included geographic or multifocal retinal dysplasia including retinal rosettes, folds, and retinal detachment, with affected dogs having a short stature, and 5/6 having hip dysplasia, though it is unclear if those latter changes are related to the same causative genetic defect (47).

## Anomalous

### Retinal dysplasia

*Retinal dysplasia* in cats is mostly associated with intrauterine viral infections which result in anomalous retinal development with anatomic features including retinal folds, rosettes, and retinal gliosis (48). This has been reported in association with natural perinatal infection with feline panleukopenia virus, and experimental infection with feline leukemia virus (FeLV) (49, 50). A case report of a kitten with retinal dysplasia reported concurrent cerebellar hypoplasia, which can be associated with perinatal feline panleukopenia infection in cats (49, 51).

### Merle ocular dysgenesis

**Figure 4:**
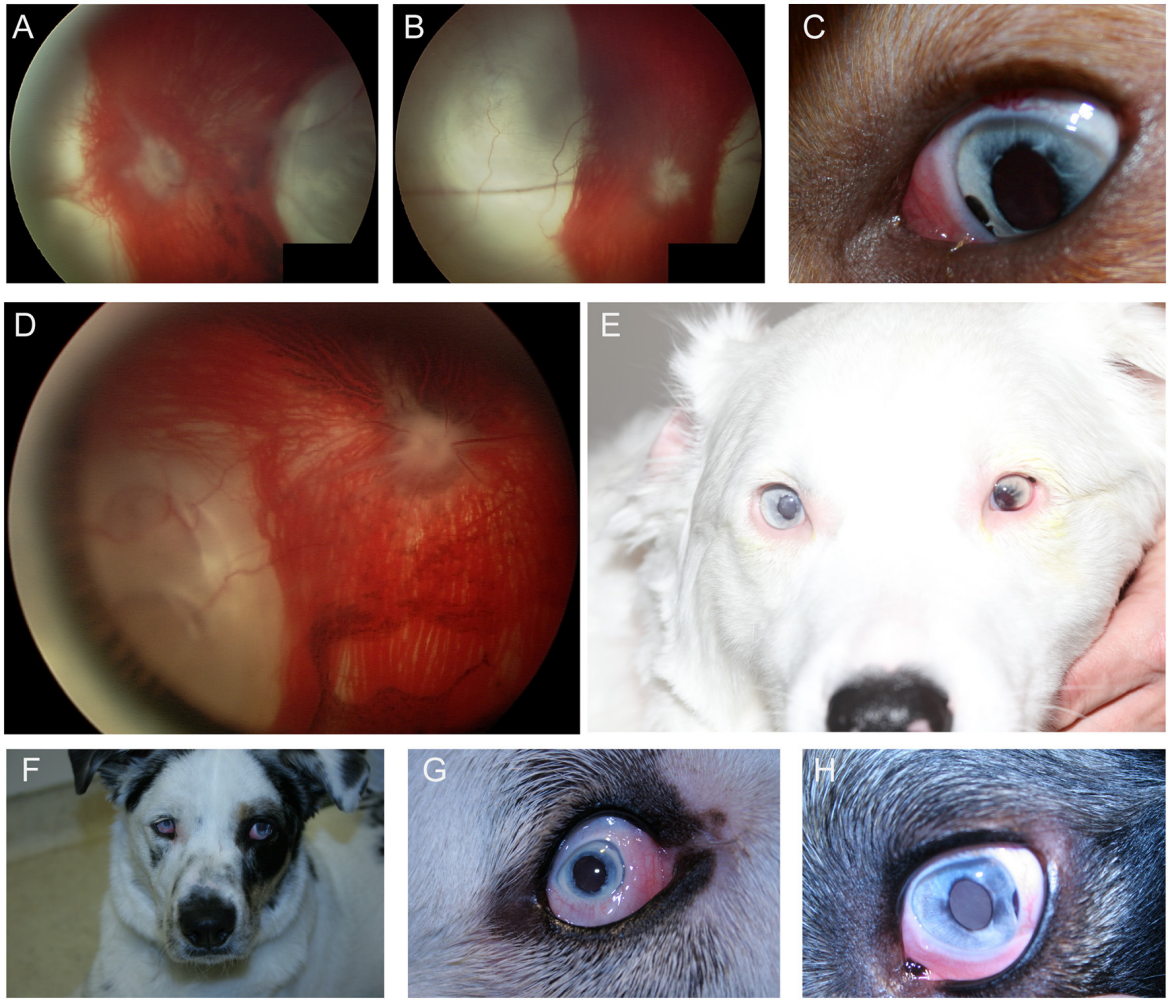
Merle ocular dysgenesis (MOD). **(A)** Right and **(B)** left fundus and **(C)** left eye of a dog affected by MOD. Both fundus images illustrate massive nasal and temporal chorioretinal colobomas. Optic disc colobomas are also present, and larger in the right optic nerve head. **(C)** An iris coloboma is seen nasally. **(D)** Severe chorioretinal coloboma and abnormal optic nerve myelination are seen in this dog's fundus. The ventral window in pigmentation is suggestive, though not diagnostic of a retinal hole. **(E)** A dog affected with severe MOD. The coat was largely white and the dog was deaf. The left eye was noticeably microphthalmic and had uveitis and hyphema. Both eyes had a cataract and the ocular fundus was not readily visible. A retinal detachment was seen ultrasonographically in the left eye offering an explanation for the source of blood. **(F)** A dog affected by MOD shows a typical merle coat. **(G)** the right eye is severely microphthalmic with a microcornea. **(H)** The left eye is microphthalmic to a lesser degree. An iris coloboma and a cataract can be seen.

In the dog, congenital ocular abnormalities are reported with the merling or dappling gene and have been termed merle ocular dysgenesis. The condition occurs in dogs affected by the merle gene which causes pigment dilution. A SINE insertion in the *Silver* pigment locus homolog (*SILV*) gene segregates with the merle phenotype, and homozygous animals are most severely affected (52). While retinal detachment and dysplasia, as well as colobomas to the choroid and optic nerve may occur with the condition there are often several ocular abnormalities as the name implies (Figure 4). Other ocular abnormalities include changes to the globe, iris and lens. Systemically, severely affected dogs will have a predominantly white coat and may show hearing deficits (Figure 4E) (52). While 25% of homozygous dogs may experience bilateral or unilateral deafness a clear association between hearing loss and ocular abnormalities has not been established for dogs (53).

## Metabolic

### Lipemia retinalis

Hyperlipoproteinemia, hyperchylomicronemia and hypertriglyceridemia (but not hypercholesterolemia alone) may result in pathognomonic peach-cream discoloration of blood seen within retinal vessels (**Figure 6A**) (54). This change is only visible in the absence of profound lipemic uveitis, as a lipid-laden aqueous may impede the ability to view the fundus of one or both eyes. This manifestation may arise due to primary or secondary hyperlipoproteinemia or hyperchylomicronemia and is reported in both dogs and cats (55, 56). The most common manifestations in cats with familial hyperchylomicronemia are fasting hyperlipemia, lipemia retinalis and peripheral neuropathy, though cutaneous xanthomas or xanthogranulomas have also been described (57, 58). Underlying pathogenesis may involve a fatty diet, genetic predisposition in miniature schnauzers or the Burmese cat, and other affected breeds, or in endocrinopathies (e.g., hypothyroidism, diabetes mellitus and hyperadrenocorticism) (54, 56). Resolution is often achieved when the underlying condition is treated and controlled. A low-fat diet and supplementation with omega-3 fatty acids are often incorporated in the treatment plan and may lead to resolution (59). Lipid-lowering medications such as bezafibrate have also been shown to be effective in dyslipidemic dogs (60). The prognosis for vision is favorable and there is no evidence that elevated serum lipids adversely affect the retina (59). Therefore, lipemia retinalis in the absence of lipemic uveitis may be an incidental finding of diagnostic utility with implications to the patients' systemic wellbeing.

### Diabetic retinopathy

Dogs with induced or naturally occurring diabetes mellitus (DM) may develop petechial retinal hemorrhages and microaneurysms, changes which are associated with diabetic retinopathy (61, 62). Oftentimes these lesions will only be seen in dogs that have undergone phacoemulsification, since diabetic cataracts tend to occur early in the disease process thereby precluding the ability to examine the fundus. In one retrospective study 21% of dogs that underwent removal of diabetic cataracts exhibited fundoscopic changes at a median time of onset of 1.4 years from the diagnosis of DM (61). Fundoscopic changes were not typically associated with clinically noticeable visual deficits, and were not shown to be associated with poor glycemic control (61). By contrast, the development of retinopathy could be inhibited with improved glycemic control in a canine model of diabetic retinopathy (63). Therefore, while dogs with diabetic retinopathy should be assessed carefully for glycemic control, this findings does not necessitate lack of glycemic control. A small longitudinal prospective study showed that 2 years following the diagnosis of DM 20% of dogs developed signs of diabetic retinopathy (64). In the same study, 55% and 64% of dogs developed systolic and diastolic hypertension, respectively, which could be a contributing factor to changes seen in the fundus of affected dogs (64). Signs of diabetic retinopathy have been reported in naturally occurring and induced DM in cats. Reports of sporadic naturally occurring DM have failed to demonstrate if affected cats had concurrent hypertension; however experimentally induced DM did result in histological microaneurysms (65, 66). Nonetheless, diabetic retinopathy is infrequent and does not seem to be of clinical significance in cats.

## Nutritional

Inappropriate nutrition may result in a variety of retinal conditions, which often present with distinct retinal manifestations. While dietary indiscretion may result in reversible *lipemia retinalis* (discussed previously), certain deficiencies can result in permanent degenerative changes in the retina.

### Taurine deficiency

Taurine deficiency in cats may result in a progressive condition of feline central retinal degeneration (FCRD, i.e., nutritional retinal degeneration) and ultimately blindness. Lesions start initially as increased granularity followed by a focal ellipsoidal region of hyperreflectivity within the area centralis and expand to two lesions which later coalesce (**Figure 6B**). If the underlying cause is corrected, the lesions do not progress, however chronic deficiency will result in a stereotypical 5-staged progression until the retina becomes fully atrophied. Historically the condition has been seen in cats fed dog food, though FCRD has become a rare condition since many dog food brands now supplement the food with taurine; nonetheless, dog food remains inappropriate for feline nutritional needs (67). With the increase in popularity of homemade, raw and vegan diets some concerns exist regarding nutritional inadequacy of noncommercial and some novel commercial diets (68–70). Cats with FCRD should undergo nutritional evaluation, as well as a cardiac evaluation to rule out dilated cardiomyopathy which may also result from taurine deficient diets.

### Vitamin A deficiency

Vitamin A deficiency is reported to result in nyctalopia (night blindness) in humans, and in some species such as cattle neonatal deficiency can cause severe ocular diseases (71). Nutritional deficiency in vitamin A is of unknown significance in the cat and dog. A mutation in retinol-binding-protein 4 (*RBP4*) reported in Irish soft-coated wheaten terriers results in vitamin A deficiency during fetal development, and subsequently in severe ocular defects such as microphthalmia, anophthalmia and coloboma (72). Previously, microphthalmia, coloboma, and other ocular abnormalities have been reported to be heritable in soft-coated wheaten terriers, and were associated with visceral developmental aberrations such as hydronephrosis and persistent right aortic arch (73).

### Vitamin E deficiency

Vitamin E deficiency in dogs manifests as pigmentary changes to the fundus due to lipofuscin accumulation and can occur with or without clinical deficits in vision. Retinal degeneration, retinal pigment epithelial dystrophy and neuroaxonal degeneration have been reported in experimental and naturally occurring vitamin E deficiency in dogs, with or without dietary insufficiency, and is suspected to be primary in English cocker spaniels and in Briards (74–79). The hallmark appearance of the fundus consists of pathognomonic small lighter brown rounded spots which correspond to lipofuscin accumulation in the retinal pigment epithelium (**Figures 6C**, **D**). The phenotype is progressive if left untreated and will ultimately result in severe visual deficits. The condition is responsive to supplementation with twice daily vitamin E (tocopherol) if treated early (77).

## Neoplastic

**Figure 5:**
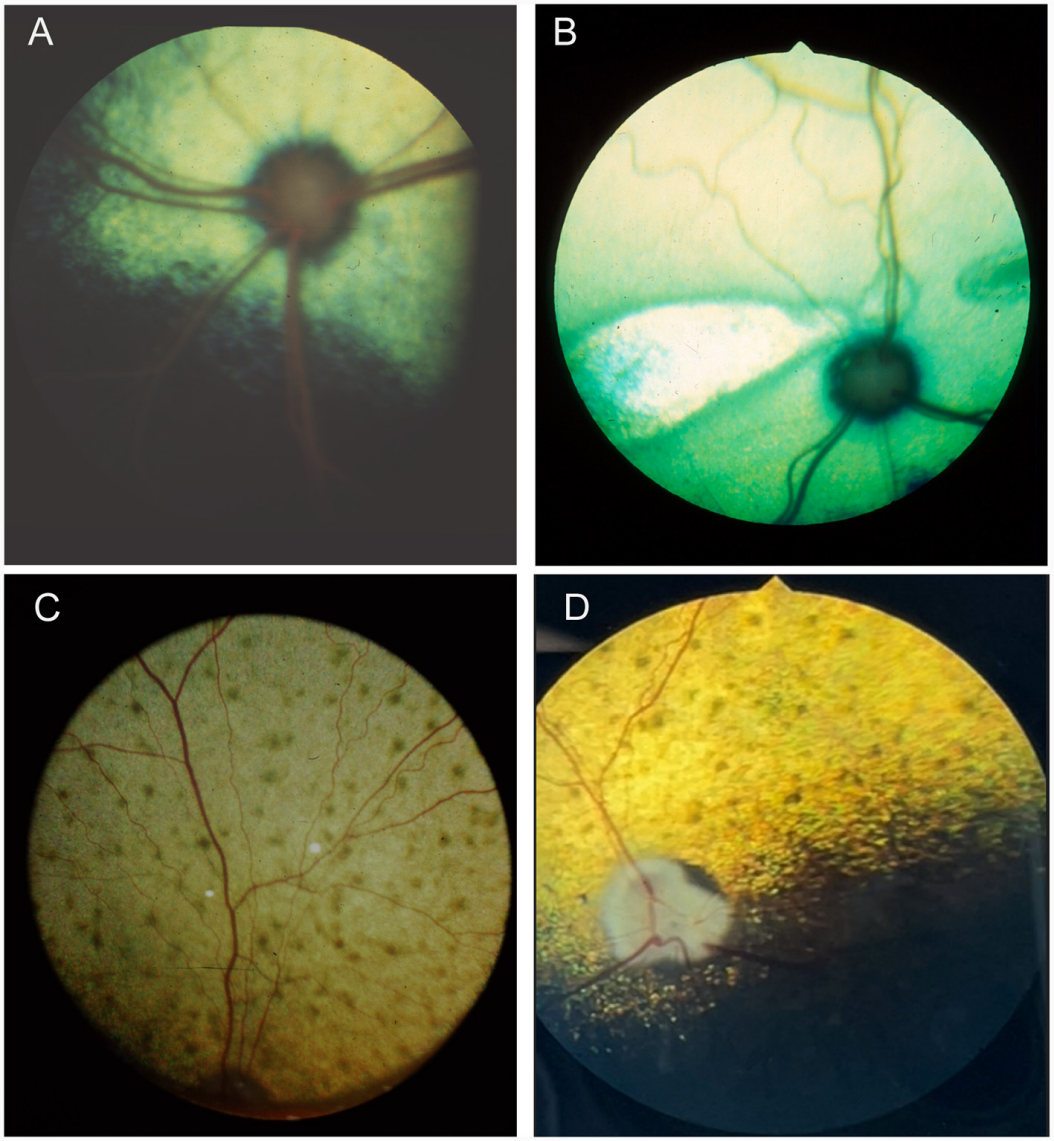
Fundic manifestations of metabolic and nutritional diseases. **(A)** Lipemia retinalis in a 4-year-old domestic shorthair cat. The retinal vasculature appears peach cream colored rather than the normal red. This cat had elevated serum cholesterol, triglycerides and chylomicronemia. Image courtesy of Sheila Crispin. **(B)** Feline central retinal degeneration (FCRD) seen in the right eye of a cat. There is an ellipsoidal region of hyperreflectivity in the temporal retina. A second smaller ellipsoidal hyperreflective lesions is present in the nasal retina. The lesions in this image correlate with Stage 3 FCRD in which two independent lesions are identified. **(C, D)** Vitamin E deficiency in the dog. **(C)** The tapetal fundus of a dog exhibits multifocal light brown rounded spots. **(D)** Multifocal punctate pigmentary spots are noted throughout the tapetal fundus. The optic nerve head is heavily myelinated and the optic nerve rim is heavily pigmented, exhibiting a likely normal variation.

Several neoplasms may result in ocular metastasis and retinal hemorrhages and uveitis are the most common manifestation of metastatic ocular disease (Figure 5A). A large-scale retrospective study found that 11.6% of 233 dogs and 13% of 100 cats with metastatic neoplasms had ocular metastases (80), with lymphoma being the commonest multicentric neoplasm in both species (Figure 5). FeLV associated T-cell lymphoma was the most common form in cats and typically presented with bilateral signs. The second most common neoplasms involving the eye were metastatic spread of mammary carcinoma in bitches (mostly unilateral), and pulmonary and squamous cell carcinoma in cats. The most frequently reported lesions affecting the fundus were choroidal involvement with subretinal hemorrhages and retinal detachment (80). Another retrospective of 173 ocular neoplasms in dogs found that 26% were metastatic or multicentric with round cell tumors including lymphoma and histiocytic sarcoma being the commonest, followed by hemangiosarcoma (81). Because of the blood-retina barrier, metastatic neoplasms that reach the subretinal space or enter through the optic nerve may be more advanced, thereby adversely affecting prognosis.

*Angioinvasive pulmonary carcinoma* with posterior segment metastasis in the cat, or feline lung-digit-syndrome, differs from other metastatic neoplasms and presents with a unique, or pathognomonic, fundoscopic appearance of wedge-shaped ischemic chorioretinopathy and unilateral or bilateral blindness (Figures 5D–K) (82). This presentation should immediately prompt the clinician to perform a comprehensive workup, and is associated with a guarded prognosis (83).

It is important to differentiate metastatic lesions in the retina from primary or solitary ocular neoplasms such as choroidal melanocytoma or meningioma, which may carry a more favorable prognosis and require a different treatment approach (*e.g.*, benign neglect, laser therapy, enucleation or with suspected extraocular extension, orbital exenteration) (Figures 5L, M).

*Cancer associated retinopathy* describes a condition in which blindness occurs without any initial funduscopic changes and is secondary to immune-mediated retinal damage, or autoimmune retinopathy. In people, the diagnosis relies on the presence of a neoplasm and circulating plasma retinal autoantibodies (84). The condition resembles sudden acquired retinal degeneration syndrome (SARDS), though differences have been reported in the profile of retinal responses to different wavelengths of light (e.g., chromatic pupillometry). The condition is reported in people and suspected in dogs (85). As with SARDS a comprehensive workup including electroretinography and advanced imaging is recommended for a diagnosis that is largely reliant on exclusion.

## Inflammatory

Inflammatory diseases that affect the fundus can be classified as infectious or non-infectious in origin. Inflammation is commonly associated with many of the conditions described and can be part of the primary disease process or can be secondary to breakdown of the blood-retina barrier.

**Figure 6:**
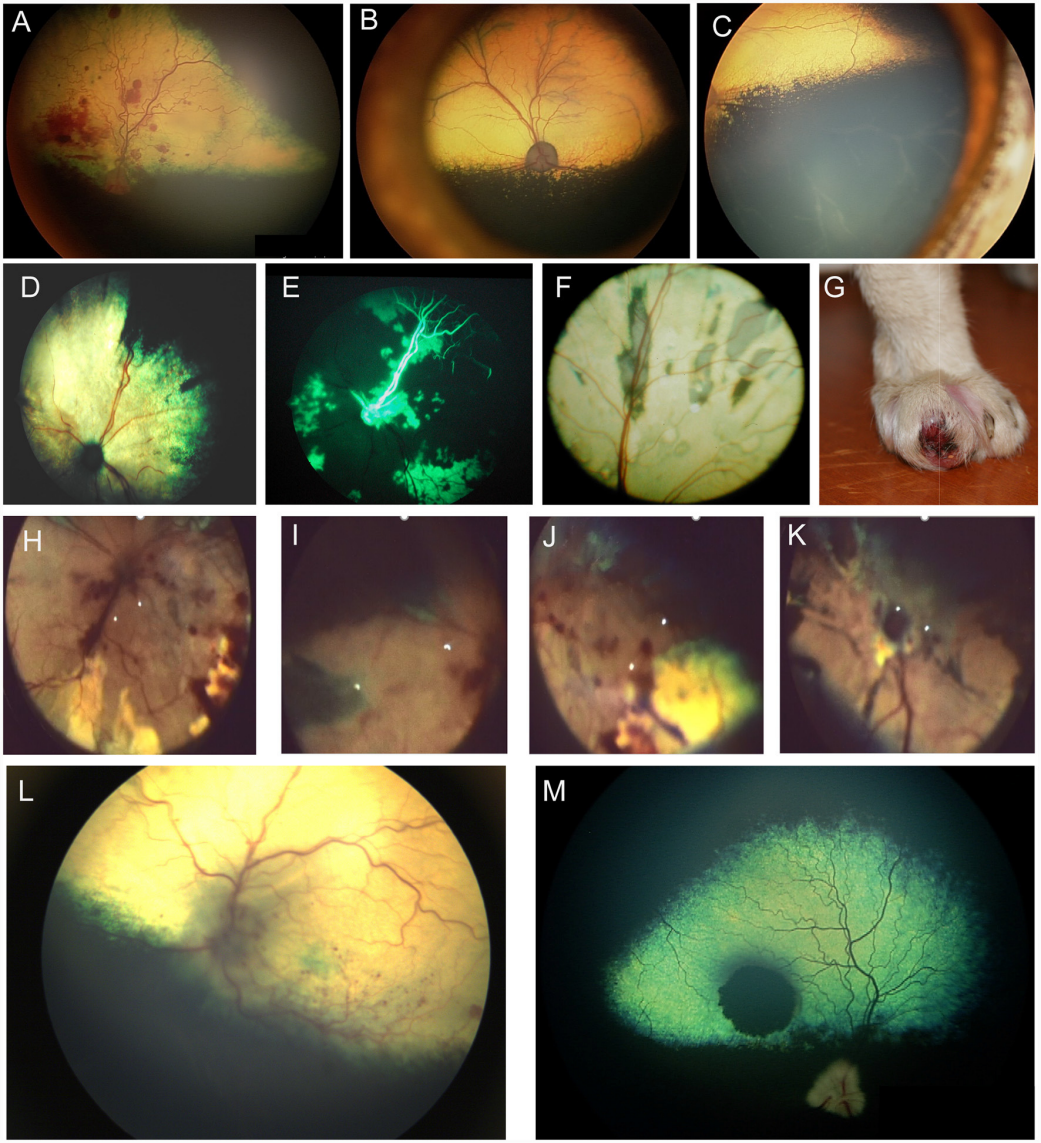
Ocular neoplasia. **(A)** Funduscopy of a 10-year-old female spayed Beagle diagnosed with stage IV B-cell lymphoma and bilateral multifocal retinal hemorrhages. **(B)** Dorsal and **(C)** ventral fundus images of a 7.5-year-old female spayed Bernese Mountain dog with bilateral perivascular cuffing suspected to be secondary to malignant histiocytosis. The patient had presented with inappetence, dysphagia and sneezing and was also diagnosed with a pulmonary mass. **(D–K)** Angioinvasive pulmonary carcinoma, i.e., lung-digit syndrome in the cat. **(D)** Wedge-shaped chorioretinal necrosis, vascular leakage and darkening of the optic nerve head consistent with ischemic damage. **(E)** Fluorescein angiography in the same eye shows filling defects along with dye leakage consistent with profound ischemic damage. **(F)** Fundus image from a cat showing radiating wedges of chorioretinal ischemic lesions and associated tapetal loss. **(G)** A digit of a feline with ocular lesions and diagnosed pulmonary carcinoma. **(H–J)** Right and **(K)** left eyes of a cat with a digit lesion and pulmonary manifestations. The retina has massive wedge-shaped lesions consistent with chorioretinal necrosis, there are multifocal retinal hemorrhages, loss of normal architecture and blackening of the optic nerve head. **(L)** Left eye of a dog diagnosed with a retrobulbar mass on CT scan. There is optic neuritis with loss of distinction of the optic nerve margins, as well as multiple punctate optic nerve and retinal hemorrhages. **(M)** Solitary choroidal melanocytic neoplasm in a dog. This lesion appeared elevated on fundoscopy.

**Figure 7:**
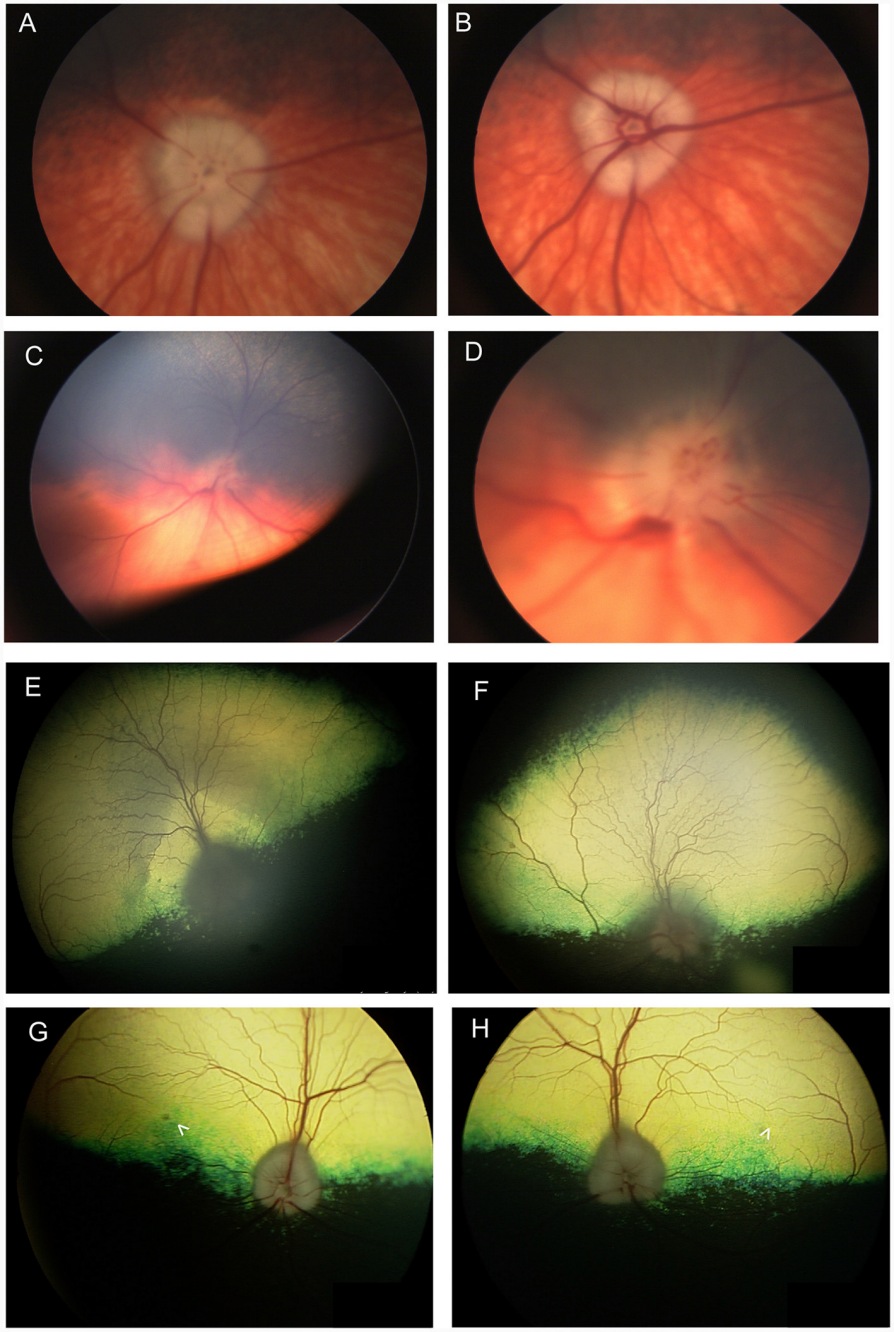
Optic nerve disease. **(A, B)** Right and **(C, D)** left eyes of a dog with optic neuritis secondary to meningoencephalitis of unknown origin. **(D)** A magnified view of **(C)** showing that the optic nerve head has indistinct margins and punctate hemorrhages. There are also hemorrhages around the optic nerve head. **(A, B)** The left optic nerve head appears swollen. An optic pit (gray focal point in the center of the nerve head) is still visible in spite of the swelling which is contrary to the common notion that pathologic optic nerve swelling is accompanied by the loss of an identifiable pit. **(A, B)** were obtained in succession and the difference between them exemplifies the change in vascular appearance with pulse phase. **(E)** Right and **(F)** left eyes of a dog presenting with bilateral blindness and optic neuritis. The optic nerve has a dull appearance with indistinct margins. In **(E)** the peripapillary region is hyperreflective while in the **(F)** left the peripapillary region is hyporeflective. **(G)** Right and **(H)** left eyes of a 9-year-old Anatolian Shepherd presenting with acute bilateral blindness and papilledema. The optic nerve heads appear bilaterally swollen, but a pit is still distinguishable. A focal round lesion is seen in the area centralis of both eyes (denoted by arrowheads). An MRI and CSF sample did not show abnormalities. The dog received a course of oral steroids and antibiotics and regained full vision, however the fundic changes remained.

In a study of 96 dogs presenting with optic neuritis, 35 were diagnosed with multifocal meningoencephalitis of unknown etiology and had other neurological abnormalities, while 42 presented with isolated optic neuritis (86). All cases presented with absent pupillary light reflex (PLR) or menace response, and in most cases both were absent. Most of the dogs in the study were treated with anti-inflammatory doses of prednisone, and for those with follow-up, 69.4% remained blind. Optic neuritis in cats has not been reported in association with immune-mediated etiologies, and is mostly associated with infectious diseases, lymphoma or rarely with systemic hypertension (87). The changes seen with optic neuritis can vary, and depend somewhat on chronicity (**Figure 7**). **Figures 7A**–**D** illustrate the lack of symmetry that may be present in the eyes of an affected individual animal. Traditionally, optic neuritis appears with loss of distinction to the margins of the optic nerve head, hemorrhages in or around the optic nerve head and changes to its color which may include hyperemia or a dull appearance once myelin has been lost (Figures 6L, 7C–F, **Figure 9F**). Other times optic neuritis may manifest as papilledema or optic nerve swelling, and be associated with blindness (Figures 7C, D, G, H). While some textbook articles state that a distinct physiologic pit is not seen in pathologic swelling (88), the latter examples help demonstrate that this may not always be the case.

## Infectious

**Figure 8:**
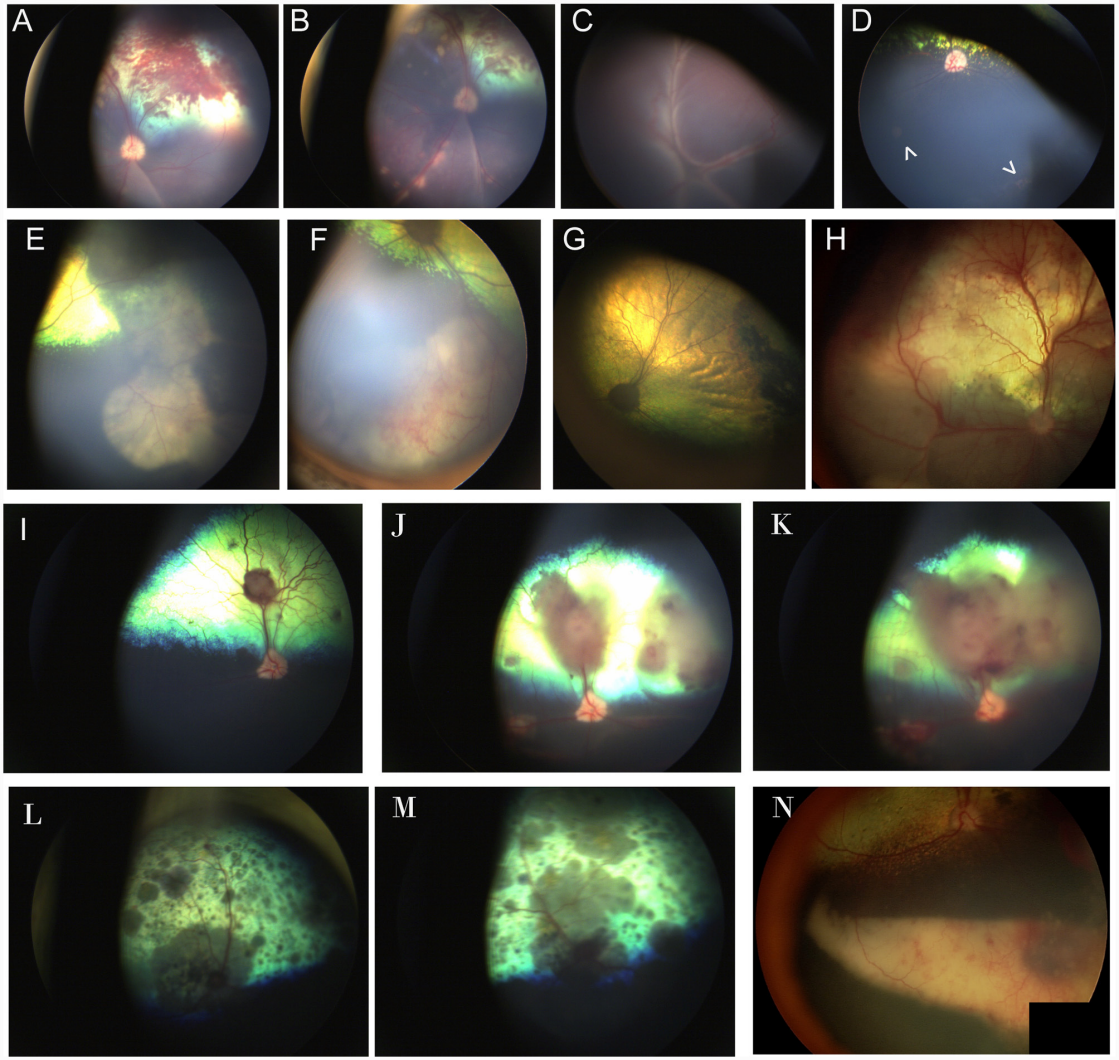
Chorioretinitis secondary to systemic mycoses. **(A, B)** Multifocal subretinal hemorrhages, retinal detachment, and tan chorioretinal granulomas in the left eye of a dog affected by systemic blastomycosis. Note how the granulomas course along the retinal vasculature. **(C)** Right and **(D)** left eyes of a dog affected by systemic blastomycosis. **(C)** Complete retinal detachment with fine perivascular infiltrates. **(D)** Two focal granulomas are noted as pale regions in the non-tapetal fundus (arrowheads). Faint cystoid peripheral atrophy can be seen ventrally and exemplifies the peripheral location in which granulomas can be found and the insidious appearance that may hinder diagnosis. **(E, F)** Large chorioretinal granulomas seen in the far periphery and non-tapetal fundus of the right eye of a dog affected by systemic blastomycosis. **(G)** 4 months following treatment with intravenous amphotericin B, there is regional tapetal hyperreflectivity and the area of prior granulomatous lesions appears as a pigmented chorioretinal scar. **(H)** Multifocal retinal hemorrhages, retinal detachment and hyporeflective inflammatory infiltrates in the eye of a dog affected by systemic blastomycosis. **(I–K)** Images of the right eye of a mixed breed dog affected by systemic *Aspergillus deflectus* confirmed by culture that was euthanized due to progression of disease in spite of an extensive hospitalization and workup along with treatment with itraconazole and amphotericin B. **(I)** At time of presentation there are several subretinal tan granulomas and associated retinal hemorrhage. **(J)** Ten days later the granulomas have progressed in size and number, and have caused focal retinal detachments. The margins of the optic nerve appear less sharp and the optic nerve head is hyperemic, suspicious for optic neuritis. **(K)** Three days following **(J)**. There is increase in size of the granulomas and retinal detachment and worsening of optic neuritis. **(L, M)** Multifocal diffuse pigmented granulomas, chorioretinitis and optic neuritis in the right **(L)** and left **(M)** eyes of a cat affected by systemic cryptococcus. **(N)** Eye of a dog affected by systemic protothecosis. There are chorioretinal pigmentary changes to the retina, a large ventral subretinal granuloma along with retinal and vitreal hemorrhages.

**Figure 9:**
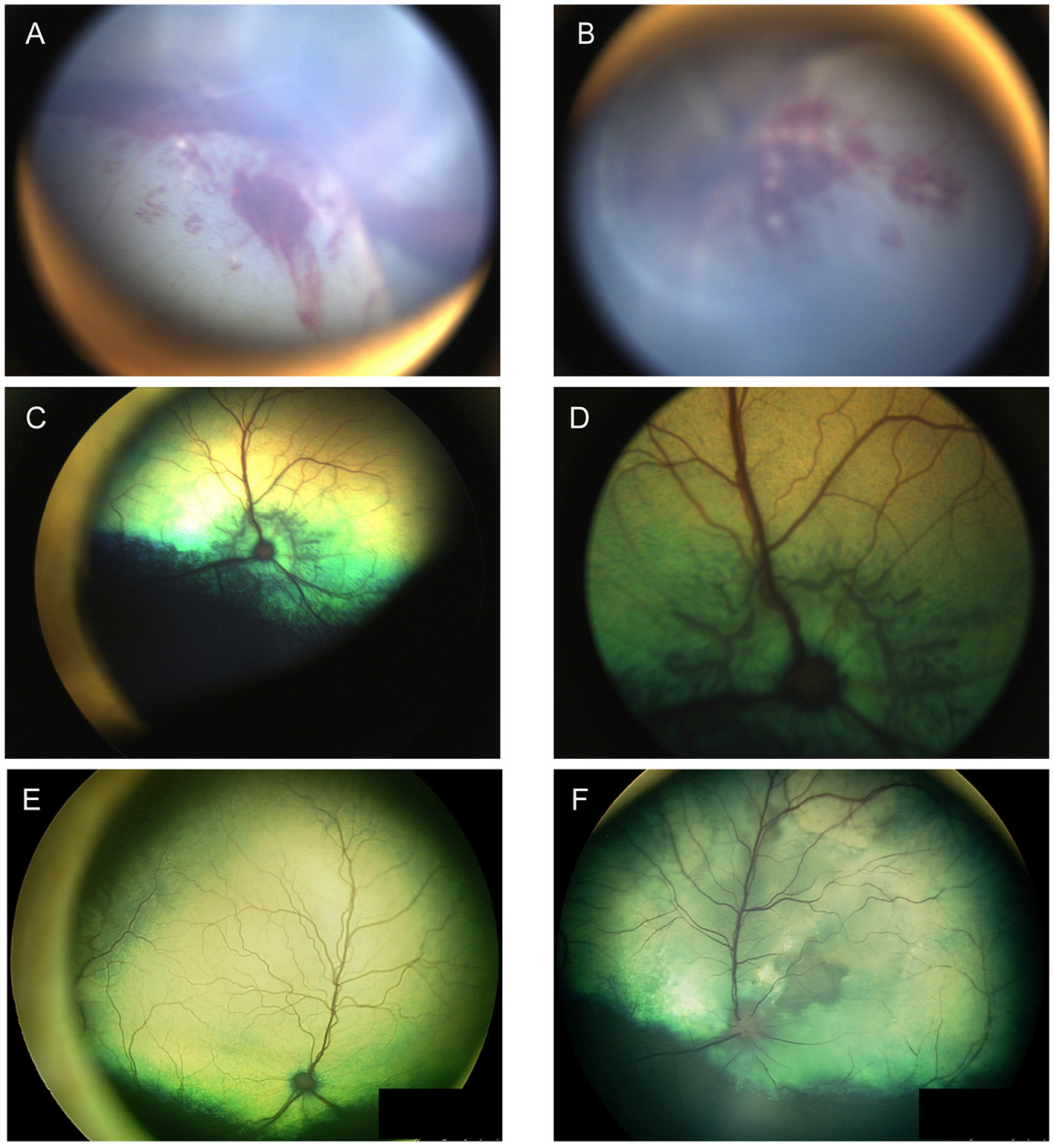
Infectious diseases. **(A)** Right and **(B)** left eyes of a mixed breed dog that was adopted from the Philippines and presented for acute blindness. The dog was found to be seropositive by IFA for *Babesia canis, Ehrlichia canis* and *Bartonella* spp. *E. canis* positivity was also confirmed by PCR and SNAP 4DX. Both eyes exhibit profound retinal detachment and intraretinal hemorrhages and granulomas. **(C)** Starburst peripapillary retinal edema and retinitis in a cat affected by feline infectious peritonitis. The cat had resolution of severe uveitis and ocular signs following treatment with GS-441524, though retinal atrophy remained. **(D)** Higher magnification of the same region as seen in **(C)** is suggestive of elevation of the peripapillary retina. **(E)** The right and **(F)** left eyes of a feline affected by toxoplasmosis with bilateral blindness and granulomatous disease in the lungs. **(E)** The retina is largely normal in appearance and blindness is attributed to a retrobulbar granuloma. **(F)** Multifocal retinal granulomas, subretinal infiltrates and optic neuritis, retinal separation and regions of tapetal hypo and hyper reflectivity are noted.

Numerous systemic infections may result in ocular manifestations; some of which affect the fundus more commonly than the anterior segment of the eye. At times, changes in the fundus are the first or only sign of those systemic conditions. Retinitis, retinal hemorrhages and chorioretinitis are common with systemic mycoses such as blastomycosis and cryptococcosis (Figure 8), as well as with rickettsial diseases (Figure 9). Those changes can be associated with infectious organisms hematogenously spreading into the retina, entry via the optic nerve or meninges, or subsequent inflammatory responses and vasculitis. Infectious diseases such as chronic monocytic ehrlichiosis in dogs or feline infectious peritonitis (FIP) may also result in profound polyclonal gammopathy, which may lead to subsequent hyperviscosity of the blood and dilation and tortuosity of retinal vasculature.

In a large study that was conducted in Brazil, 849 eyes of 428 cats that died of systemic infectious disease were evaluated by necropsy, 29% of which manifested with histologic ocular disease (89). The most important ocular diseases included FeLV, FIP and *cryptococcus* which often manifested with uveitis, optic neuritis and meningitis. Coinfection of FeLV and feline immunodeficiency virus (FIV) along with *cryptococcus* was identified in a small subset of tissues using immunohistochemistry.

### Systemic mycoses

The prevalence of systemic mycosis is highly dependent on climate and environment (90). Therefore, when considering the likelihood of an underlying fungal infection the reader must consider the geographical location of the patient. Climate change among other causes contribute to a certain degree of flux in the geographic distribution of infectious organisms. When unexpected cases present both autochthonous and heterochthonus infections must be considered. The reader is referred to a recent review by Ashraf *et al*. for comprehensive global maps detailing the endemic distribution of common fungal infections that affect various species (91).

Systemic mycoses occur more frequently in dogs than in cats, with *Cryptococcus neoformans* and *Histoplasma capsulatum* being the commonest in cats. *Cryptococcus neoformans* and *gattii* cause infection via inhalation and may spread to the eye via hematogenous dissemination, or through the optic nerve, and cause chorioretinitis (92). The disease predominates in the posterior segment but can extend to the anterior segment causing uveitis and keratic precipitates (93). Granulomas and variably pigmented chorioretinal lesions are commonly seen on fundic examination (Figures 8L, M). Though uncommon, *Cryptococcus* may result in intraocular disease and chorioretinitis in the dog (94, 95).

Like many other fungal infections *Coccidioides* spp. causes infection via inhalation followed by hematogenous dissemination to various organs. In dogs, coccidioidomycosis may cause ocular signs as the only manifestation of disease, and those can be either bilateral or unilateral (96). A granulomatous uveitis is the most common ocular manifestation, though disease is also present in the retina, and chorioretinitis and retinal detachments may be noted (96). Infections with coccidioidomycosis are uncommon in cats and may be difficult to diagnose via cytology. In a retrospective study of 48 cats with systemic coccidioidomycosis 13% presented with anterior uveitis or retinal detachment, 44% with fever, inappetence and weight loss, 56% with dermatologic signs, and 25% with respiratory signs (97).

A study evaluating causes of panuveitis in dogs in the United-States Midwest found that blastomycosis was the second most common cause after presumed immune-mediated disease, with histoplasmosis and aspergillosis occurring less commonly (98). A study of 73 dogs with ocular blastomycosis showed that most of the dogs had posterior segment disease with or without endophthalmitis, and only six eyes had anterior segment disease (99). Ocular lesions can include chorioretinitis and variably sized chorioretinal granulomas (Figures 8A–H). The prognosis for blastomycosis is good except with severe involvement of the lungs or involvement of the central nervous system (100). Cases with ocular disease may also have a poor outcome, and in one report half the cases with ocular involvement died, though some responded to amphotericin B (99, 101). The severity of blastomycosis with intraocular involvement, the inability of the eye to effectively clear the organism and inadequate penetration of itraconazole may reduce prognosis and affect the treatment plan, at times necessitating expensive treatment with intravenous amphotericin B or enucleation of the affected eye (100). It is therefore important to include a thorough exam of the fundus in animals diagnosed with blastomycosis. In a study looking at 35 cats affected by blastomycosis 21 had reported ocular abnormalities and yet only 18 of the cats in this series had been examined by a veterinary ophthalmologist (102). Bilateral ocular lesions were present in 15/19 cats included in the study, 10/19 cats had ocular inflammatory lesions and 9/19 had ocular manifestations of central nervous system disease including cortical blindness, absent PLR, anisocoria or Horner's syndrome. Inflammatory changes in the posterior segment included vitritis, vitreal hemorrhage, chorioretinitis, retinal detachment and retinal hemorrhage (102).

Histoplasmosis is the second most common fungal infection in cats following cryptococcosis. After the organisms are inhaled, they disseminate, and the animals develop a multisystemic disease with nonspecific signs. In a study of 55 cats affected by ocular histoplasmosis 45 of the cats had signs of chorioretinitis including chorioretinal granulomas and retinal detachments, and 32 cats were blinded by the lesions (103). 19/45 cats presented with anterior uveitis, and cataracts were seen in 11/45 affected cats, secondary glaucoma was rare and was diagnosed in 2/55 cats (103). A study of 79 affected dogs reported ocular signs in only three, and those included chorioretinitis and retinal detachment, however it was not specified if all animals underwent an ocular exam (104).

While rare, incidental infections with other systemic mycosis affecting the retina of dogs include acremoniasis (105), aspergillosis (98, 106) (Figures 8I–K), geotrichosis (107), pseudallescheriasis (108) and candidiasis (109). German shepherd dogs are predisposed to disseminated mycosis with *Aspergillus* spp., *Pseudallescheria* spp. and other fungal organisms (110, 111). While infections with the aforementioned have been reported to have ocular involvement in cats, retinal involvement was only reported with candidiasis (112). Immune suppression may contribute to sporadic infections and has been reported in association with some of the aforementioned cases.

*Algal disease- Prototheca wickerhamii* and *P. zopfi* infections have been reported both in dogs and cats, though ocular manifestations appear to be rare in cats. *Prototheca* has a worldwide distribution (except Antarctica), though infections are more common in warm humid climates. Clinical signs usually include hemorrhagic diarrhea, blindness, ataxia and pyuria (113, 114). In the eye, the algae most often causes uveitis, granulomatous chorioretinitis, and retinal detachment and blindness occurs in ~50% of dogs (Figure 8N) (113, 115). The organisms may be sampled from the choroid, though diagnosis is often obtained from aspirates or biopsies of cutaneous lesions, rectal scrapings or urine culture and cytology (114, 116). Combination therapy may be superior to monotherapy, and treatment often involves anti-fungal agents and antibiotics, though definitive treatment guidelines have not been formulated for the disease (114, 117). While the prognosis is generally considered grave, extended survival has been reported (115). Acute blindness in dogs with recent acute hemorrhagic diarrhea should prompt the clinician to include protothecosis as a differential diagnosis.

#### Tick-borne

Retinal lesions may accompany tick-borne illnesses as a manifestation of their systemic effects. The symptoms in the fundus vary in frequency, with non-specific hemorrhages being the commonest sign. The most common rickettsial organisms which are associated with retinal manifestations include *Ehrlichia* spp., *Anaplasma* spp., and *Rickettsia rickettsia* (Figures 9A, B). Exudative retinal detachment is the most common change seen with *Ehrilichia canis*, though optic neuritis, uveitis and hemorrhages associated with thrombocytopenia or inflammation are often reported (118). *Anaplasma phagocytophila* and other rickettsial diseases have been sporadically associated with chorioretinitis and retinal detachment (119). Retinal lesions seen with *R. rickettsii* (Rocky Mountain Spotted Fever) resemble those seen with canine ehrlichiosis with chorioretinitis and vasculitis also reported (120, 121). There are sporadic reports of chorioretinitis associated with *Bartonella vinsonii* and *Borrelia burgorferi* (Lyme borreliosis) (122, 123). Diagnosis is often made via PCR or serology, and tests should be submitted for multiple pathogens if tick-borne disease is suspected, due to the likelihood of co-infection with multiple organisms, and the non-specific ocular signs (124–126). Tetracyclines are the treatment of choice for most rickettsial and other tick-borne diseases, though profound ocular inflammation or bone marrow suppression (as manifested by thrombocytopenia in chronic disease) may warrant judicious use of steroids (126–128). Early treatment may permit retinal reattachment if a separation occurred. Topical ophthalmic medication is indicated for anterior segment disease but may not adequately penetrate to the posterior segment. Chronic inflammation and retinal detachment may result in secondary glaucoma and complete retinal detachment may be an indication for prophylactic anti-glaucoma medication. *Babesia* spp., are tick-borne protozoa that often accompany infections with rickettsial organisms (125). While less frequently reported in the literature, *Babesia* spp. have been associated with profound posterior segment disease in the dog, including complete retinal detachment (129). Babesiosis may be challenging to treat, often requiring combinations of anti-protozoal medications such as imidocarb, and anti-protozoal antibiotics such as clindamycin or azithromycin (130).

*Cytauxzoon felis* is a tick-transmitted protozoan which causes an often-fatal disseminated disease in cats. Due to the severity of non-ocular disease, findings from eyes are seldom reported in affected cats, however a small histological case series illustrated that schizont-laden macrophages may be present in the retina associated with retinal detachment and panuveitis (131).

#### Bacterial

*Mycobacterium*- Mycobacteria have been reported to cause ocular disease in a variety of species. Feline mycobacteriosis is a zoonotic condition that has a geographical distribution in the UK. In cats, ocular lesions were reported in 6.6% of affected animals (132). However, a recent study from the UK found that ocular presentation without systemic involvement occurs in 24% of affected cats (133). When ocular involvement is present, the posterior segment is most severely affected with granulomatous to pyogranulomatous chorioretinitis and retinal detachment noted in most affected cats (133). The study demonstrated that remission was able to be achieved for those 8 cats that were treated with a prolonged course (5 months or more) combining three antibiotics (rifampicin, pradofloxacin and azithromycin or clarithromycin), with or without enucleation or incisional biopsy.

*Bartonella* spp. species are small gram-negative bacteria that are known causative agents of endocarditis, myocarditis and granulomatous disease (134). Involvement of *Bartonella* spp. in ocular disease has been suspected in both dogs (122) and cats (135), but chorioretinitis was only reported in dogs. Its role as a primary causative agent has been a source of controversy among clinicians. Since prolonged treatment, often with multiple medications is required, a diagnosis is typically made after comprehensive workup and exclusion of more commonly recognized causative agents.

Rare reports of other causes of bacterial uveitis exist in dogs, including brucellosis and leptospirosis. While chorioretinitis was reported with dogs infected by *Brucella canis* anterior segment disease may preclude evaluation of the posterior segment in certain cases (136). Panuveitis and serous retinal detachment was reported in one dog that presented with active leptospirosis based on convalescent titers (98).

#### Viral

*Feline infectious peritonitis* (FIP) is a severe viral disease that is caused by feline coronavirus (FCoV), the disease may present in various forms that have been divided to effusive (wet) and noneffusive (dry) or to visceral and neurological. Ocular FIP is considered a neurological form of FIP that is usually noneffusive and is commonly associated with a poorer prognosis. Affected cats present with lethargy, weight loss and may exhibit severe neurological changes (137). Ocular signs of FIP often include severe fibrinous anterior uveitis with mutton-fat keratic precipitates. When the ocular media is sufficiently clear to visualize the fundus then vascular cuffing, optic neuritis, chorioretinitis, retinal hemorrhages and detachments are commonly seen (Figures 9C, D) (137). A massive lymphoplasmacytic inflammation may affect all ocular tissues, and macrophages dominate granulomas when present (138). While historically the disease has been considered to be uncurable and cats were given a guarded prognosis, treatment with adenosine nucleoside analogs shows promise in curing even the most severe forms of FIP, though access to these medications is limited (139).

The virus that causes *Feline panleukopenia* has affinity to rapidly dividing cells, therefore ocular changes are only seen in cats affected before 3 months of age, or *in utero*. As discussed above, infected kittens may present with retinal dysplasia, retinal atrophy and optic nerve hypoplasia, with or without cerebellar lesions (49).

*Canine herpes virus* (CHV) has been reported to cause severe ocular systemic disease in both experimental and naturally occurring infections in dogs. While naturally occurring disease is associated with severe conjunctivitis and ocular surface disease (140), retinal pathology has been reported only with experimental infection of newborns and included retinal dysplasia, retardation of retinal maturation and necrosis (141).

The hallmark of *Canine distemper virus* is an acute catarrhal conjunctivitis with respiratory signs. Nonetheless, up to 80% of affected dogs also suffer from retinochoroiditis which is most easily identified as pigment loss and edema in the non-tapetal fundus, with or without perivascular infiltrates (142). Neurologic signs result from affinity of the virus to gray and white matter, and blindness or visual impairment may be sequalae of optic nerve or optic tract demyelination (143). While no treatment is available for canine distemper virus, its prevalence has largely diminished due to vaccination.

#### Protozoal

Protozoal diseases, including toxoplasmosis, neosporosis and leishmaniasis, are reported in cats, and less frequently in dogs. While the cat is the definitive host for *Toxoplasma*, the dog is the definitive host for *Neospora*, and the reservoir host for *Leishmania*. *Toxoplasma gondii* may induce lesions in any part of the eye, but chorioretinal lesions and anterior uveitis are the most common, and are frequent in infected animals (Figures 9E, F) (144). Histologically or clinically the retina may exhibit intraretinal inflammatory infiltrates, subretinal inflammatory exudation, or retinal detachment in severe cases. Diagnosis is usually made by serology (including both IgG, and IgM titers), though PCR testing from ocular samples can also be performed. Four-weeks of treatment with clindamycin and trimethoprim-sulfonamide are usually needed, though complete clearance of the parasite is unlikely (145). Retinal changes which occur with *N. caninum* often include mild retinitis of the inner retina, and chorioretinitis with occasional tissue cysts with intralesional tachyzoites in severe infections. Diagnosis is achieved by serum immunofluorescence, antibody titers, IgG agglutination or PCR (146). Treatment using clindamycin or other drugs is only partially effective (146). Co-infection of *Neospora* and *Toxoplasma*, is not infrequently reported, warranting a thorough workup of suspected cases. Leishmaniasis is most often caused by *L. infantum*, though other serovars have been reported. While ocular changes are commonly associated with *L. infantum* in the dog, those are most likely to involve the ocular adnexa and anterior segment; nonetheless, up to 12.5% of dogs diagnosed with leishmaniasis are reported to have retinal abnormalities such as chorioretinitis (147). Similar changes are reported, albeit rarely, in affected cats. Diagnosis is made by serology, PCR and cytology and remission is achieved by combination therapy with long-term combination of meglumine antimonite and allopurinol (148).

#### Parasitic

**Figure 10:**
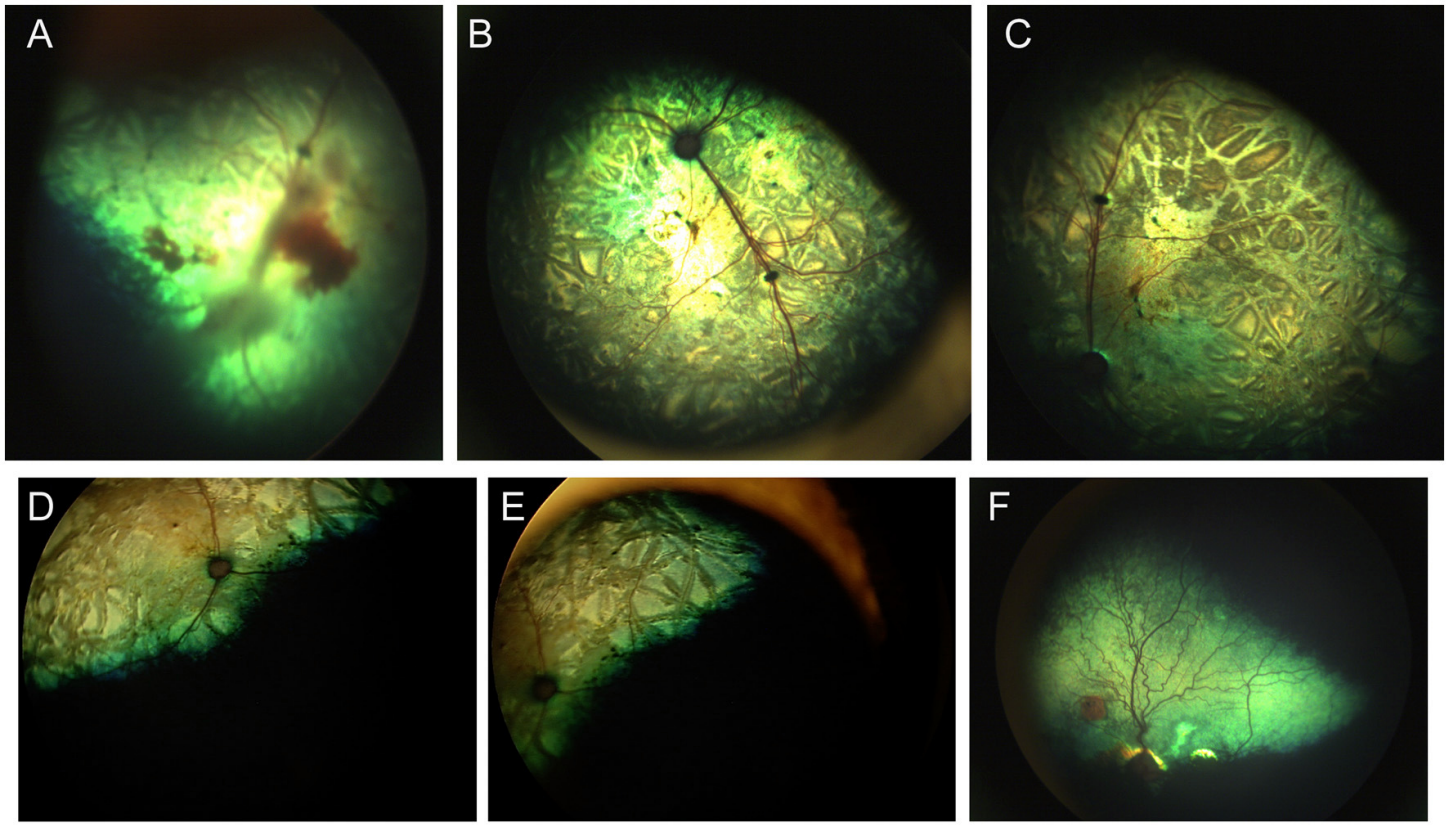
Parasitic ocular disease. **(A)** Fundus of the right eye of a 3-month-old kitten affected by larval migrans interna posterior before and **(B, C)** 1 month after a Cuterebra larva was removed from her skin. Notice the crisscrossing lines in the retina, retinal hyperreflectivity and vitreal and retinal hemorrhages. The fellow eye was unaffected. **(D, E)** Right eye of a cat diagnosed larval migrans interna posterior. Crisscrossing and coalescing chorioretinal scars are consistent with historic infection. **(F)** Fundus of a greyhound dog with multiple hyperreflective chorioretinal scars. One circular lesion has a pigmented center which is consistent with reports of DUSN. While not proven to have a parasitic etiology, a historic parasitic exposure is suspected in many such cases.

Ophthalmomyiasis interna posterior and ocular nematodiasis secondary to ocular larval migrans has been reported in both cats and dogs. Prognosis for sight is poor to guarded, and prognosis for globe retention is variably affected by the degree of damage caused by larval migration. *Cuterebra* spp. of the order *Diptera* are the most commonly reported in the cat and a case suspected to be from the order *Diptera* was reported in the dog (149, 150). Cases of successful removal of *Cuterebra* larva from the anterior segment and the vitreous have been reported in dogs, making it a likely culprit in this species (151, 152). The animals may present asymptomatic or with lethargy and inappetence; funduscopically there are pathognomonic curvilinear cross-hatching tracts in the retina (Figures 10A–E) (149, 150). Hemorrhages, edema or the presence of actual larvae may indicate an active disease process.

*Toxocara canis* is an important gastrointestinal parasite which may cause ocular larval migrans via somatic migration. While reports of this parasitic infection are rare, funduscopic lesions consistent with *T. canis*, were found in 39% of working sheep dogs in New Zealand, and in some cases *T. canis* was confirmed histologically (153). In contrast to crisscrossing lesions seen with Cuterebra migrans, lesions associated with *T. canis* include well delineated multifocal subretinal granulomas and retinal degeneration (153). The lesions are usually not associated with active or live larval migration, however a fecal should be performed to rule out active systemic infection. In rare cases, chorioretinitis and retinal hemorrhages have been reported with *Angiostrongylus vasorum* (154). Recently, a commentary and review of Diffuse Unilateral Subacute Neuropathy (i.e., DUSN), had detailed typical patchy retinal lesions more commonly seen in male dogs as being associated with ocular larval migrans, and accidental entry of nematodes into the canine eye (155). The lesions were characterized by focal or multifocal patches of tapetal hyperreflectivity with a dark center or non-tapetal patchy depigmentation (Figure 10F). *T. canis* involvement in DUSN was reported by Johnson (156). *Toxocara canis*, and *T. cati* were the most suspected in dogs, cats and humans. Though *Baylisascaris procyonis* was commonly implicated in humans and animals, it has not been reported in the eyes of dogs and cats. Both in sheepdogs and in border collies an association was made to a diet which included raw frozen mutton and pork scraps (153, 156). As *T. canis* larva can survive for weeks in frozen tissue, it is possible that the increase in propensity of “raw” frozen diets has resulted in the increase of DUSN cases encountered clinically (155).

*Encephalitozoon cuniculi* is a spore forming intracellular microsporidium that has been most associated with phakitis in rabbits, with sporadic reports in other species (157, 158). The organism is presumed to invade the lens *in utero*, and later causes lens capsule rupture with subsequent induced uveitis and organism invasion to other ocular tissues (157). Affected rabbits may present with severe neurological symptoms (157). A case series of three young dogs reported on multiple ocular lesions including chorioretinitis, uveitis and focal cataracts (159). Disease was confirmed in the affected dogs via serum antibody testing, PCR and immunohistochemistry, and dogs were responsive to anti-inflammatory treatment and fenbendazole (159). Population studies of American household dogs found a high *E. cuniculi* seroprevalence of 21%, with young dogs more commonly affected (160). While some reports of involvement of *E. cuniculi* in canine neurological disease exist, a study exploring its involvement found it to be of limited clinical significance (161).

## Immune-mediated

A retrospective study found that the most common cause of panuveitis in dogs was idiopathic or immune-mediated disease (71%), with partial or complete serous or bullous retinal detachment being the most common posterior segment clinical sign (95% affected) (98). Dogs with immune-mediated panuveitis had more favorable outcomes when compared to dogs with infectious panuveitis. Of the dogs with immune-mediated panuveitis 9% were diagnosed as having uveodermatologic syndrome. Most dogs in the study were treated with oral prednisone (80%) for an average of 94 days as well as doxycycline hyclate (67%) for an average of 22 days and topical prednisolone acetate (80%) for an average of 191 days.

*Uveodermatologic syndrome* is a presumed immune-mediated disorder in which macrophages attack melanocytes resulting in profound uveal inflammation and pigment dispersion. The dermatological hallmarks of the condition include loss of pigment of the skin (i.e., vitiligo) and hair (i.e., leukotrichia) with the muzzle and eyelids most commonly affected (162). While the most common ocular clinical sign is uveitis, retinal detachment has been reported in 46% of dogs and choroidal depigmentation and infiltrates in 20% of dogs (162). Akitas and arctic breed dogs such as Siberian husky and Samoyed are overrepresented, though the condition may affect dogs of any breed. The early non-specific ocular signs often precede the pathognomonic dermatological signs complicating diagnosis in crossbred dogs, or dogs of underrepresented breeds. The condition is variably controlled with aggressive topical and systemic anti-inflammatory and immune suppressive treatment protocols, however vision loss is common secondary to chronic inflammation and subsequent glaucoma (162).

Idiopathic immune-mediated conditions include Sudden Acquired Retinal Degeneration Syndrome (SARDS), cancer associated retinopathy (discussed previously) and autoimmune retinopathy. Affected dogs present with largely normal retinal morphology, sudden blindness and abolished electroretinographic responses. Changes such as decreased outer retinal thickness, and increased inner retinal thickness may be noticeable using optical coherence tomography (163). Early in the disease, chromatic pupillometry usually reveals disrupted photoreceptor function and absent PLRs to a red wavelength of light with preserved pupil constriction driven by blue wavelength light stimulation of melanopsin in intrinsically photosensitive retinal ganglion cells (164). A systemic workup is recommended to rule out underlying systemic diseases and treatment options are controversial and may require prolonged systemic immune suppression to spare or restore vision (164). Non-ocular changes associated with SARDS bear clinical similarity to hyperadrenocorticism (HAC) or pituitary-dependent hypercortisolism (PDH) in dogs. Clinical signs of HAC, systemic hypertension, and proteinuria were commonly found in dogs with SARDS, with sex hormone elevations present in 85% of dogs diagnosed with SARDS, and cortisol elevations present in 69% of dogs (165). A recent study found that urine MT6s:creatinine ratio was higher in dogs with PDH compared to dogs with SARDS, but not compared to normal dogs (166).

## Ischemic

Ischemic retinal changes may occur following major disruption to vascular supply. In most cases, animals may present with other major systemic illnesses, acute glaucoma, or following trauma. As described above (Figures 6D–K) ischemic retinal disease develops in cats with angioinvasive metastatic pulmonary carcinoma, or lung-digit syndrome (82). Clusters of neoplastic cells occlude major choroidal vessels resulting in wedges of choroidal ischemia and retinal degeneration, along with retinal hemorrhages. The differentiation between glaucoma, trauma or feline metastatic neoplasm is usually straightforward and the diagnosis will guide the recommended treatment plan. While ischemic damage to the retina will often cause severe and irreversible visual deficits, there has been increased interest in the use of neuroprotective agents to spare retinal ganglion cell function (10).

## Toxic retinopathy

A variety of drugs and toxins can result in retinal damage and blindness. Perhaps most important to mention is the toxic effect of systemically administered fluoroquinolones, and particularly enrofloxacin in cats, which while rare, can cause permanent blindness even when using the recommended dose (167). Age and renal disease are considered risk factors. Enrofloxacin-associated retinal toxicity results in retinal degeneration and vascular attenuation (167). While all systemic fluoroquinolones should be used with caution, to date there are no reports of any retinal toxicity associated with the new fluoroquinolone pradofloxacin (168, 169). Orbifloxacin has been reported to cause retinal toxicity at 18 times the recommended dose (170) and marbofloxacin has been anecdotally reported to cause retinal toxicity (168).

Of note, ivermectin may cause idiosyncratic blindness in dogs, horses and cats which may not present with fundoscopic lesions but exhibit visual impairment and decreased electroretinographic function (indicative of a loss of photoreceptor function) (171–173). Two affected dogs presented with blindness and mild obtundation, and ocular exam revealed retinal edema and retinal folds (174). One dog presented with optic neuritis (86). Cats and dogs with defects in the *ABCB1* gene (formerly *MDR1*) may present with severe concurrent signs of neurotoxicity when exposed to macrocyclic lactones (e.g., avermectins and milbemycins) (171, 172, 175). The *ABCB1-1*Δ mutation is typically seen in herding-type breeds, primarily but not limited to collies, Shetland sheepdogs, Australian shepherds and crosses of affected breeds (175). Genetic testing is readily available for dogs and should be performed in predisposed breeds to determine a safe antiparasitic treatment plan (175). The condition may result in permanent blindness, though improvement is reported with supportive care, removal of the toxin is achieved by cessation or via intravenous lipid emulsion therapy (173, 174, 176).

## Traumatic

Severe trauma to the eye and head may result in optic nerve ischemia, retinal hemorrhages, or detachments. Affected patients will likely have a history of a traumatic episode or other signs of trauma noted to the body and eye. Hyphema and hemophthalmia will preclude a fundic exam in severe ocular trauma (177).

## Vascular

### Retinal hemorrhages

**Figure 11:**
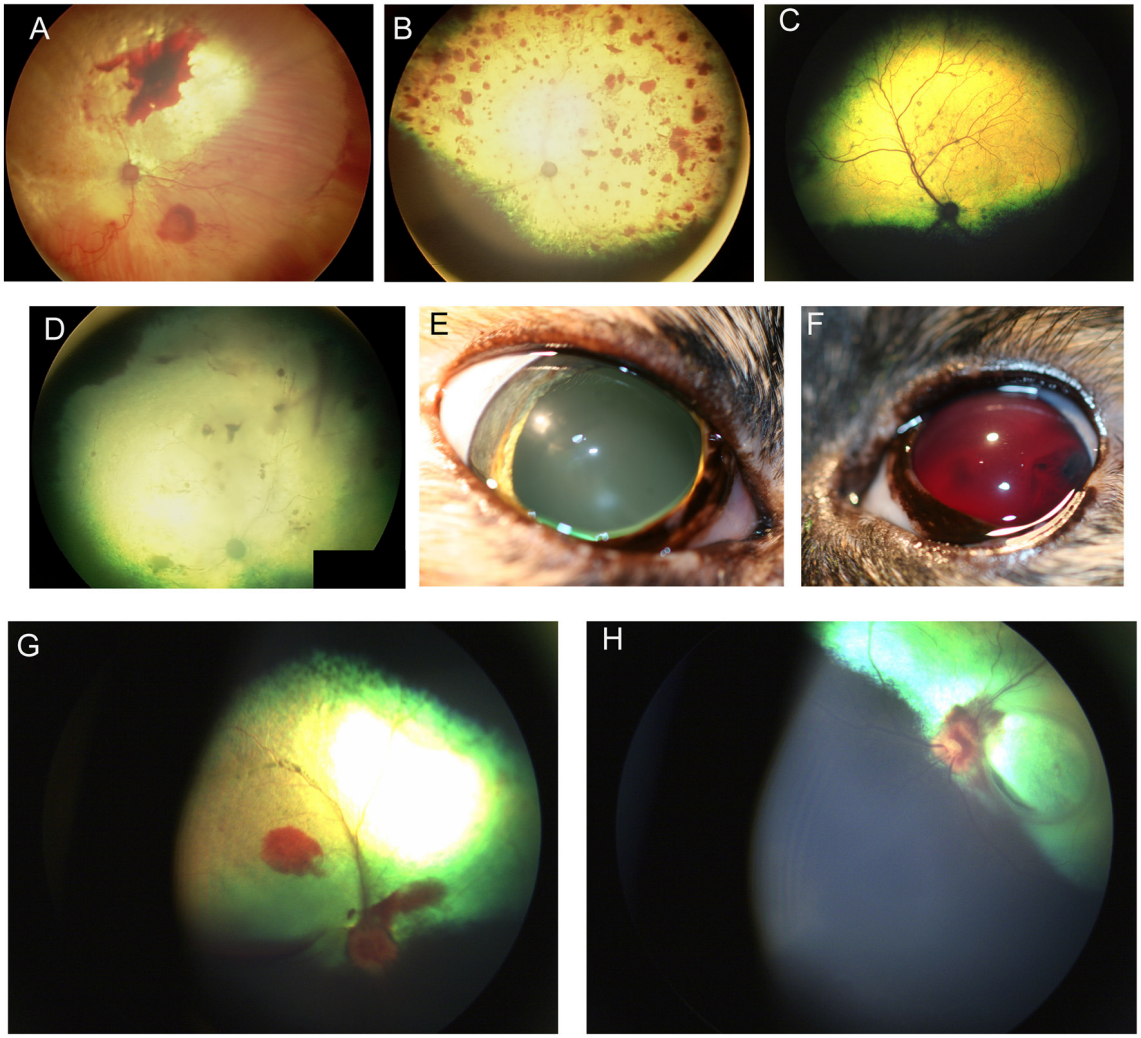
Retinal hemorrhages. **(A)** Subalbinotic fundus of the right eye of a cat manifesting with subretinal and intraretinal (punctate) hemorrhages of unknown etiology. Tapetal hyperreflectivity and vascular attenuation are also visible. **(B–F)** Feline hypertensive retinopathy. **(B)** Multifocal retinal hemorrhages ranging in size from punctate to one optic nerve in diameter, along with tapetal hyperreflectivity and vascular attenuation. Upon presentation the patient had a systolic blood pressure of 215 mmHg and a hematocrit of 20 and a prolonged aPTT at 89.6s (Ref 23.6–54.9s). The cat later became blind and recumbent. Humane euthanasia was elected and a necropsy showed chronic renal failure and a cerebellar hemorrhage. **(C)** Multifocal punctate retinal hemorrhages and retinal lesions. This cat was diagnosed with a functional adrenal carcinoma and secondary hyperaldosteronism and a systolic blood pressure of 300 mmHg. **(D)** Right fundus, **(E)** right eye, and **(F)** left eye of a cat suffering from hypertensive retinopathy in the right eye, and hypertensive oculopathy in the left. **(D)** the fundic vasculature is greatly attenuated and the retina is hyporeflective. There are punctate intra-retinal hemorrhages, subretinal and vitreal hemorrhages. **(E)** the pupil is dilated and non-responsive to light. **(F)** Hyphema and blood clots are formed in the anterior chamber. **(G)** Optic neuritis, subretinal and vitreal hemorrhage in the right eye of a dog affected by immune mediated thrombocytopenia. **(H)** Optic neuritis with flame shaped hemorrhages tracking along the nerve fiber layer, subretinal hemorrhages and focal retinal atrophy in the left eye of the same dog.

Retinal hemorrhages are nonspecific signs of retinal disease that occur when vascular endothelial permeability permits egress of red blood cells. This can occur from hypoxemic damage, vasculitis, increase in blood pressure or other conditions (Figure 11). While a variety of conditions may manifest in retinal hemorrhages (e.g., anemia, thrombocytopenia, coagulopathies), the most common underlying cause of retinal hemorrhages is systemic hypertension. Often these hemorrhages may be incidental findings, but their importance cannot be understated, as they can facilitate the timely identification of life-threatening hypertension or systemic illness. Hemorrhage appearance may indicate the location or retinal layer in which they occur: punctate hemorrhages often manifest intraretinal hemorrhages which are restricted in spread, flame shaped hemorrhages track the fibers of the nerve fiber layer, keelboat hemorrhages are epiretinal allowing a gravity line to form. Subretinal hemorrhage can be extensive and bright red, while choroidal hemorrhages are often darker in appearance and have indistinct margins (Figures 11A–D, G, H). In chronic hypertensive retinopathy one might see changes in retinal vessel morphology such as increased tortuosity or aneurysms (often called “box-carring”), and patchy retinal degeneration, edema, or detachment. One study demonstrated that 62% of hypertensive dogs (systolic blood pressure >160 mmHg) presented with at least 1 ocular sign (178). Visual deficits are common in hypertensive cats and may be associated with either primary or secondary hypertension (179). While permanent blindness occurs in affected cats, favorable prognosis for vision is possible even for eyes with complete retinal detachment, if treated early (180). Another study that looked at punctate retinal hemorrhages in dogs found that 60% of 83 affected dogs suffered from concurrent systemic diseases (181).

### Vascular dilation and tortuosity

The presence of vascular tortuosity and dilation should prompt the clinician to measure blood pressure and perform a general workup including a complete blood count and total protein evaluation to rule out hypertension or hyperviscosity. Hyperviscosity and vascular engorgement has been reported secondary to infectious diseases that cause poly or monoclonal gammopathy, and more infrequently due to increased cellular burden from conditions such as multiple myeloma and polycythemia vera (182–185). Segmentation of the blood column or “box-car” appearance may be seen both with hyperviscosity and hypertensive retinopathy. One clinical sign that may help distinguish the underlying cause relates to the affected vasculature- in cases of hypertension the arterial vasculature is affected, while the venous vasculature is spared, and in cases of hyperviscosity both venous and arterial vasculature are affected. Retinal changes seen with hyperviscosity and systemic hypertension may also include retinal edema, retinal detachment and hemorrhages as discussed earlier in the text.

### Anemia and thrombocytopenia

Ocular lesions were reported in 42% of dogs affected by thrombocytopenia with or without concurrent anemia and the severity and prevalence of the lesions was correlated to the severity of thrombocytopenia (186). The most common changes in the fundus included retinal hemorrhages and retinal detachment, ocular lesions were not associated with anemia in that study (Figures 11G, H) (186). In cats, anemic retinopathy has been reported in association with chronic and severe anemia, most often due to *haemobartonella felis* or FeLV and manifests with superficial and deep retinal hemorrhages and pale thin retinal vessels; vascular aneurysms or sacculations were also reported (187, 188). Rarely vascular congestion and dilation has been reported with left sided congestive heart failure in the dog (143).

## Summary of conclusion

In summary, retinal and fundic changes in the dog and cat can offer a wealth of information with regards to patient health. While many changes are non-specific, some are pathognomonic or highly suggestive of specific disease processes and can expedite the path to a correct diagnosis and appropriate medical plan. Familiarity with the wide range of normal variations, as well as the ability to distinguish active from static lesions is paramount. Clinicians are encouraged to incorporate the fundic exam during wellness evaluations and evaluation of sick patients.

## Author contributions

SP-J: Data curation, Funding acquisition, Resources, Supervision, Validation, Writing – review & editing. BB-C: Conceptualization, Data curation, Funding acquisition, Investigation, Resources, Validation, Visualization, Writing – original draft, Writing – review & editing.

## References

[1] Iqbal U. Smartphone fundus photography: a narrative review. Int J Retina Vitreous. (2021) 7:44. 10.1186/s40942-021-00313-9 34103075 PMC8186054

[2] Occelli LM Daruwalla A De Silva SR Winkler PA Sun K Pasmanter N. A large animal model of RDH5-associated retinopathy recapitulates important features of the human phenotype. Hum Mol Genet. (2021). 10.1093/hmg/ddab316 34726233 PMC9029234

[3] Winkler PA Occelli LM Petersen-Jones SM. Large animal models of inherited retinal degenerations: a review. Cells. (2020) 9:882. 10.3390/cells9040882 32260251 PMC7226744

[4] Hildebrandt F Benzing T Katsanis N. Ciliopathies. N Engl J Med. (2011) 364:1533–43. 10.1056/NEJMra1010172 21506742 PMC3640822

[5] Makelainen S Hellsand M van der Heiden AD Andersson E Thorsson E Haggstrom J. Deletion in the bardet-biedl syndrome gene ttc8 results in a syndromic retinal degeneration in dogs. Genes. (2020) 11:1090. 10.3390/genes11091090 32962042 PMC7565673

[6] Hitti-Malin RJ Burmeister LM Lingaas F Kaukonen M Pettinen I Lohi H. A missense variant in the bardet-biedl syndrome 2 gene (BBS2) leads to a novel syndromic retinal degeneration in the shetland sheepdog. Genes. (2021) 12:1771. 10.3390/genes12111771 34828377 PMC8624581

[7] Chew T Haase B Bathgate R Willet CE Kaukonen MK Mascord LJ. A coding variant in the gene bardet-biedl syndrome 4 (BBS4) is associated with a novel form of canine progressive retinal atrophy. G3 (Bethesda). (2017) 7:2327–35. 10.1534/g3.117.043109 28533336 PMC5499139

[8] Acland GM Aguirre GD Ray J Zhang Q Aleman TS Cideciyan AV. Gene therapy restores vision in a canine model of childhood blindness. Nat Genet. (2001) 28:92–5. 10.1038/ng0501-92 11326284

[9] Occelli LM Zobel L Stoddard J Wagner J Pasmanter N Querubin J. Development of a translatable gene augmentation therapy for CNGB1-retinitis pigmentosa. Mol Ther. (2023) 31:2028–41. 10.1016/j.ymthe.2023.04.005 37056049 PMC10362398

[10] Hopper RG Montiani-Ferreira F da Silva Pereira J Fritz MC Ruggiero VJ Sapienza JS. Presumed neuroprotective therapies prescribed by veterinary ophthalmologists for canine degenerative retinal and optic nerve diseases. Vet Ophthalmol. (2021) 24:229–39. 10.1111/vop.12878 33682296 PMC8137575

[11] Comander J Weigel DiFranco C Sanderson K Place E Maher M. Natural history of retinitis pigmentosa based on genotype, vitamin A/E supplementation, and an electroretinogram biomarker. JCI Insight. (2023) 8:167546. 10.1172/jci.insight.167546 37261916 PMC10445682

[12] Shaffer LG. Special issue on canine genetics: animal models for human disease and gene therapies, new discoveries for canine inherited diseases, and standards and guidelines for clinical genetic testing for domestic dogs. Hum Genet. (2019) 138:437–40. 10.1007/s00439-019-02025-5 31056728

[13] Bradbury AM Gurda BL Casal ML Ponder KP Vite CH Haskins ME. review of gene therapy in canine and feline models of lysosomal storage disorders. Hum Gene Ther Clin Dev. (2015) 26:27–37. 10.1089/humc.2015.002 25671613 PMC4516914

[14] Katz ML Rustad E Robinson GO Whiting REH Student JT Coates JR. Canine neuronal ceroid lipofuscinoses: promising models for preclinical testing of therapeutic interventions. Neurobiol Dis. (2017) 108:277–87. 10.1016/j.nbd.2017.08.017 28860089 PMC5675811

[15] Hubler M Haskins ME Arnold S Kaser-Hotz B Bosshard NU Briner J. Mucolipidosis type II in a domestic shorthair cat. J Small Anim Pract. (1996) 37:435–41. 10.1111/j.1748-5827.1996.tb02444.x 8887204

[16] Murray JA Blakemore WF Barnett KC. Ocular lesions in cats with GM1-gangliosidosis with visceral involvement. J Small Anim Pract. (1977) 18:1–10. 10.1111/j.1748-5827.1977.tb05818.x 404469

[17] Cork LC Munnell JF Lorenz MD. The pathology of feline GM2 gangliosidosis. Am J Pathol. (1978) 90:723–34. 415617 PMC2018239

[18] Cuddon PA Higgins RJ Duncan ID Miller SP Parent JM Moser AB. Polyneuropathy in feline niemann-pick disease. Brain. (1989) 112:1429–43. 10.1093/brain/112.6.1429 2557121

[19] Brown DE Thrall MA Walkley SU Wenger DA Mitchell TW Smith MO. Feline Niemann-Pick disease type C. Am J Pathol. (1994) 144:1412–5. 8203477 PMC1887453

[20] Blakemore WF. A case of mannosidosis in the cat: clinical and histopathological findings. Journal of Small Animal Practice. (1986) 27:447–55. 10.1111/j.1748-5827.1986.tb03962.x

[21] Haskins ME Aguirre GD Jezyk PF Desnick RJ Patterson DF. The pathology of the feline model of mucopolysaccharidosis I. Am J Pathol. (1983) 112:27–36. 6407329 PMC1916323

[22] Haskins ME Aguirre GD Jezyk PF Patterson DF. The pathology of the feline model of mucopolysaccharidosis VI. Am J Pathol. (1980) 101:657–74. 6778219 PMC1903664

[23] Bildfell R Matwichuk C Mitchell S Ward P. Neuronal ceroid-lipofuscinosis in a cat. Vet Pathol. (1995) 32:485–8. 10.1177/030098589503200505 8578638

[24] Katz ML Coates JR Cooper JJ O'Brien DP Jeong M Narfstrom K. Retinal pathology in a canine model of late infantile neuronal ceroid lipofuscinosis. Invest Ophthalmol Vis Sci. (2008) 49:2686–95. 10.1167/iovs.08-1712 18344450

[25] Friend SC Barr SC Embury D. Fucosidosis in an English springer spaniel presenting as a malabsorption syndrome. Aust Vet J. (1985) 62:415–20. 10.1111/j.1751-0813.1985.tb14124.x 3833197

[26] Keller CB Lamarre J. Inherited lysosomal storage disease in an English springer-spaniel. J Am Vet Med Assoc. (1992) 200:194–5. 10.2460/javma.1992.200.02.194 1559875

[27] Traas AM Wang P Ma X Tittiger M Schaller L O'Donnell P. Correction of clinical manifestations of canine mucopolysaccharidosis I with neonatal retroviral vector gene therapy. Mol Ther. (2007) 15:1423–31. 10.1038/sj.mt.6300201 17519893

[28] Jolly RD Johnstone AC Norman EJ Hopwood JJ Walkley SU. Pathology of mucopolysaccharidosis IIIA in Huntaway dogs. Vet Pathol. (2007) 44:569–78. 10.1354/vp.44-5-569 17846229

[29] Ellinwood NM Wang P Skeen T Sharp NJ Cesta M Decker S. A model of mucopolysaccharidosis IIIB (Sanfilippo syndrome type IIIB): N-acetyl-alpha-D-glucosaminidase deficiency in Schipperke dogs. J Inherit Metab Dis. (2003) 26:489–504. 10.1023/A:1025177411938 14518829

[30] Perez ML Kridel HA Gallagher A Sheppard BJ Reese S Kondo H. Mucopolysaccharidosis type VI in a juvenile miniature schnauzer dog with concurrent hypertriglyceridemia, necrotizing pancreatitis, diabetic ketoacidosis. Can Vet J. (2015) 56:272–7. 25750448 PMC4327141

[31] Haskins ME Aguirre GD Jezyk PF Schuchman EH Desnick RJ Patterson DF. Mucopolysaccharidosis type VII (Sly syndrome). Beta-glucuronidase-deficient mucopolysaccharidosis in the dog. Am J Pathol. (1991) 138:1553–5. 1905109 PMC1886403

[32] Alroy J Orgad U DeGasperi R Richard R Warren CD Knowles K. Canine GM1-gangliosidosis. A clinical, morphologic, histochemical, and biochemical comparison of two different models. Am J Pathol. (1992) 140:675–89. 1546746 PMC1886155

[33] Kolicheski A Johnson GS Villani NA O'Brien DP Mhlanga-Mutangadura T Wenger DA. GM2 Gangliosidosis in Shiba Inu Dogs with an In-Frame Deletion in HEXB. J Vet Inter Med. (2017) 31:1520–6. 10.1111/jvim.14794 28833537 PMC5598891

[34] Tomatsu S Pitz S Hampel U. Ophthalmological findings in mucopolysaccharidoses. J Clin Med. (2019) 8:1467. 10.3390/jcm8091467 31540112 PMC6780167

[35] Wilkerson MJ Lewis DC Marks SL Prieur DJ. Clinical and morphologic features of mucopolysaccharidosis type II in a dog: naturally occurring model of Hunter syndrome. Vet Pathol. (1998) 35:230–3. 10.1177/030098589803500311 9598589

[36] Prakalapakorn SG Proia AD Yanovitch TL DeArmey S Mendelsohn NJ Aleck KA. Ocular and histologic findings in a series of children with infantile pompe disease treated with enzyme replacement therapy. J Pediatr Ophthalmol Strabismus. (2014) 51:355–62. 10.3928/01913913-20140813-01 25139343 PMC4413912

[37] Walvoort HC. Glycogen storage disease type II in the Lapland dog. Vet Q. (1985) 7:187–90. 10.1080/01652176.1985.9693981 3901497

[38] Coussa RG Roos JC Aroichane M Miron MC Ospina LH. Progression of retinal changes in Gaucher disease: a case report. Eye (Lond). (2013) 27:1331–3. 10.1038/eye.2013.180 23970031 PMC3831135

[39] Hartley WJ Blakemore WF. Neurovisceral glucocerebroside storage (Gaucher's disease) in a dog. Vet Pathol. (1973) 10:191–201. 10.1177/030098587301000302 4798391

[40] Brownstein S Meagher-Villemure K Polomeno RC Little JM. Optic nerve in globoid leukodystrophy (Krabbe's disease). Ultrastructural changes. Arch Ophthalmol. (1978) 96:864–70. 10.1001/archopht.1978.03910050466015 418756

[41] Bradbury AM Bagel JH Nguyen D Lykken EA Pesayco Salvador J Jiang X. rabbe disease successfully treated via monotherapy of intrathecal gene therapy. J Clin Invest. (2020) 130:4906–20. 10.1172/JCI133953 32773406 PMC7456224

[42] Bundza A Lowden JA Charlton KM. Niemann-Pick disease in a poodle dog. Vet Pathol. (1979) 16:530–8. 10.1177/030098587901600504 573013

[43] Kuwamura M Awakura T Shimada A Umemura T Kagota K Kawamura N. Type C niemann-pick disease in a boxer dog. Acta Neuropathol. (1993) 85:345–8. 10.1007/BF00227733 8460536

[44] Goldstein O Guyon R Kukekova A Kuznetsova TN Pearce-Kelling SE Johnson J. COL9A2 and COL9A3 mutations in canine autosomal recessive oculoskeletal dysplasia. Mammal Genome. (2010) 21:398–408. 10.1007/s00335-010-9276-4 20686772 PMC2954766

[45] Carrig CB MacMillan A Brundage S Pool RR Morgan JP. Retinal dysplasia associated with skeletal abnormalities in Labrador Retrievers. J Am Vet Med Assoc. (1977) 170:49–57. 830631

[46] Iwabe S Dufour VL Guzman JM Holle DM Cohen JA Beltran WA. Focal/multifocal and geographic retinal dysplasia in the dog-In vivo retinal microanatomy analyses. Vet Ophthalmol. (2020) 23:292–304. 10.1111/vop.12725 31746146 PMC7071990

[47] Rodarte-Almeida AC Petersen-Jones S Langohr IM Occelli L Dornbusch PT Shiokawa N. Retinal dysplasia in American pit bull terriers–phenotypic characterization and breeding study. Vet Ophthalmol. (2016) 19:11–21. 10.1111/vop.12243 25522758

[48] MacMillan A. Retinal dysplasia in the dog and cat. Vet Clin North Am Small Anim Pract. (1980) 10:411–5. 10.1016/S0195-5616(80)50037-9 6998097

[49] Percy DH Scott FW Albert DM. Retinal dysplasia due to feline panleukopenia virus infection. J Am Vet Med Assoc. (1975) 167:935–7. 1184424

[50] Albert DM Lahav M Colby ED Shadduck JA Sang DN. Retinal neoplasia and dysplasia. I Induction by feline leukemia virus. Invest Ophthalmol Vis Sci. (1977) 16:325–37. 191424

[51] Kilham L Margolis G Colby ED. Cerebellar ataxia and its congenital transmission in cats by feline panleukopenia virus. J Am Vet Med Assoc. (1971) 158::888. 5103504

[52] Clark LA Wahl JM Rees CA Murphy KE. Retrotransposon insertion in SILV is responsible for merle patterning of the domestic dog. Proc Natl Acad Sci U S A. (2006) 103:1376–81. 10.1073/pnas.0506940103 16407134 PMC1360527

[53] Strain GM Clark LA Wahl JM Turner AE Murphy KE. Prevalence of deafness in dogs heterozygous or homozygous for the merle allele. J Vet Intern Med. (2009) 23:282–6. 10.1111/j.1939-1676.2008.0257.x 19192156

[54] Crispin S. Ocular lipid deposition and hyperlipoproteinaemia. Prog Retin Eye Res. (2002) 21:169–224. 10.1016/S1350-9462(02)00004-6 12062534

[55] Jones BR Wallace A Harding DR Hancock WS Campbell CH. Occurrence of idiopathic, familial hyperchylomicronaemia in a cat. Vet Rec. (1983) 112:543–7. 10.1136/vr.112.23.543 6879969

[56] Crispin SM. Lipids and the eye. Vet J. (2016) 212:90–8. 10.1016/j.tvjl.2016.03.015 27117400

[57] Johnstone AC Jones BR Thompson JC Hancock WS. The pathology of an inherited hyperlipoproteinaemia of cats. J Comp Pathol. (1990) 102:125–37. 10.1016/S0021-9975(08)80118-1 2324336

[58] Jones BR Johnstone AC Cahill JI Hancock WS. Peripheral neuropathy in cats with inherited primary hyperchylomicronaemia. Vet Rec. (1986) 119:268–72. 10.1136/vr.119.11.268 3022456

[59] Xenoulis PG Steiner JM. Canine hyperlipidaemia. J Small Anim Pract. (2015) 56:595–605. 10.1111/jsap.12396 26456868

[60] De Marco V Noronha KSM Casado TC Nakandakare ER Florio JC Santos EZ. Therapy of canine hyperlipidemia with bezafibrate. J Vet Intern Med. (2017) 31:717–22. 10.1111/jvim.14701 28382723 PMC5435059

[61] Landry MP Herring IP Panciera DL. Funduscopic findings following cataract extraction by means of phacoemulsification in diabetic dogs: 52 cases (1993-2003). J Am Vet Med Assoc. (2004) 225:709–16. 10.2460/javma.2004.225.709 15457664

[62] Hausler HR Sibay TM Campbell J. Retinopathy in a dog following diabetes induced by growth hormone. Diabetes. (1964) 13:122–6. 10.2337/diab.13.2.122 14127423

[63] Engerman RL Kern TS. Progression of incipient diabetic retinopathy during good glycemic control. Diabetes. (1987) 36:808–12. 10.2337/diabetes.36.7.808 3556280

[64] Herring IP Panciera DL Werre SR. Longitudinal prevalence of hypertension, proteinuria, and retinopathy in dogs with spontaneous diabetes mellitus. J Vet Intern Med. (2014) 28:488–95. 10.1111/jvim.12286 24417733 PMC4858021

[65] Hatchell DL Toth CA Barden CA Saloupis P. Diabetic retinopathy in a cat. Exp Eye Res. (1995) 60:591–3. 10.1016/S0014-4835(05)80074-0 7615025

[66] Linsenmeier RA Braun RD McRipley MA Padnick LB Ahmed J Hatchell DL. Retinal hypoxia in long-term diabetic cats. Invest Ophthalmol Vis Sci. (1998) 39:1647–57. 9699554

[67] Aguirre GD. Retinal degeneration associated with the feeding of dog foods to cats. J Am Vet Med Assoc. (1978) 172:791–6. 640940

[68] Wilson SA Villaverde C Fascetti AJ Larsen JA. Evaluation of the nutritional adequacy of recipes for home-prepared maintenance diets for cats. J Am Vet Med Assoc. (2019) 254:1172–9. 10.2460/javma.254.10.1172 31039096

[69] Zafalon RVA Risolia LW Vendramini THA Ayres Rodrigues RB Pedrinelli V Teixeira FA. Nutritional inadequacies in commercial vegan foods for dogs and cats. PLoS One. (2020) 15:e0227046. 10.1371/journal.pone.0227046 31951617 PMC6968870

[70] Davies RH Lawes JR Wales AD. Raw diets for dogs and cats: a review, with particular reference to microbiological hazards. J Small Anim Pract. (2019) 60:329–39. 10.1111/jsap.13000 31025713 PMC6849757

[71] Donkersgoed JV Clark EG. Blindness caused by hypovitaminosis A in feedlot cattle. Can Vet J. (1988) 29:925–7. 17423169 PMC1680936

[72] Kaukonen M Woods S Ahonen S Lemberg S Hellman M Hytonen MK. Maternal inheritance of a recessive RBP4 defect in canine congenital eye disease. Cell Rep. (2018) 23:2643–52. 10.1016/j.celrep.2018.04.118 29847795 PMC6546432

[73] Van der Woerdt A Stades J Boeve M. Multiple ocular anomalies in two related litters of soft coated wheaten terriers. Vet Comp Ophthalmol. (1995) 5:5.

[74] Riis RC Sheffy BE Loew E Kern TJ Smith JS. Vitamin E deficiency retinopathy in dogs. Am J Vet Res. (1981) 42:74–86. 7224322

[75] Davidson MG Geoly FJ Gilger BC McLellan GJ Whitley W. Retinal degeneration associated with vitamin E deficiency in hunting dogs. J Am Vet Med Assoc. (1998) 213:645–51. 10.2460/javma.1998.213.05.645 9731258

[76] McLellan GJ Elks R Lybaert P Watte C Moore DL Bedford PG. Vitamin E deficiency in dogs with retinal pigment epithelial dystrophy. Vet Rec. (2002) 151:663–7. 10.1136/vr.151.22.663 12498409

[77] McLellan GJ Bedford PG. Oral vitamin E absorption in English Cocker Spaniels with familial vitamin E deficiency and retinal pigment epithelial dystrophy. Vet Ophthalmol. (2012) 2:48–56. 10.1111/j.1463-5224.2012.01049.x 22831287

[78] Bedford PG. Retinal pigment epithelial dystrophy in the Briard. Vet Rec. (2009) 164:377. 10.1136/vr.164.12.377-a 19305014

[79] Lightfoot RM Cabral L Gooch L Bedford PG Boulton ME. Retinal pigment epithelial dystrophy in Briard dogs. Res Vet Sci. (1996) 60:17–23. 10.1016/S0034-5288(96)90124-1 8745249

[80] Bandinelli MB Viezzer Bianchi M Wronski JG Santos de Mello L Blanco DeMartini R Savi C. Ophthalmopathologic characterization of multicentric or metastatic neoplasms with an extraocular origin in dogs and cats. Vet Ophthalmol. (2020) 23:814–27. 10.1111/vop.12803 32687655

[81] Krieger EM Pumphrey SA Wood CA Mouser PJ Robinson NA Maggio F. Retrospective evaluation of canine primary, multicentric, and metastatic intraocular neoplasia. Vet Ophthalmol. (2022). 10.1111/vop.12988 35395124

[82] Cassotis NJ Dubielzig RR Gilger BC Davidson MG. Angioinvasive pulmonary carcinoma with posterior segment metastasis in four cats. Vet Ophthalmol. (1999) 2:125–31. 10.1046/j.1463-5224.1999.00068.x 11397254

[83] Goldfinch N Argyle DJ. Feline lung-digit syndrome: unusual metastatic patterns of primary lung tumours in cats. J Feline Med Surg. (2012) 14:202–8. 10.1177/1098612X12439267 22370862 PMC10822433

[84] Goldstein SM Syed NA Milam AH Maguire AM Lawton TJ Nichols CW. Cancer-associated retinopathy. Arch Ophthalmol. (1999) 117:1641–5. 10.1001/archopht.117.12.1641 10604671

[85] Grozdanic SD Lazic T Kecova H Mohan K Adamus G Kuehn MH. Presumed cancer-associated retinopathy (CAR) mimicking Sudden Acquired Retinal Degeneration Syndrome (SARDS) in canines. Vet Ophthalmol. (2021) 24:125–55. 10.1111/vop.12853 33369040 PMC8048582

[86] Smith SM Westermeyer HD Mariani CL Gilger BC Davidson MG. Optic neuritis in dogs: 96 cases (1983-2016). Vet Ophthalmol. (2018) 21:442–51. 10.1111/vop.12528 29251394

[87] Nell B Optic neuritis in dogs and cats. The Veterinary clinics of North America. Small Animal Pract. (2008) 38:403–15. 10.1016/j.cvsm.2007.11.005 18299014

[88] McLellan GJ. Diseases of the Canine Optic Nerve. in:Gelatt KN Gilger BSG Hendrix BC Kern TJP, editor. Veterinary Ophthalmology Sixth Edition. Hoboken, NJ: John Wiley & Sons, Inc. (2021).

[89] Wronski JG de Cecco BS Raiter J Henker LC de Lorenzo C Bandinelli MB. Ophthalmic and immunopathological characterization of systemic infectious diseases in cats. Vet Pathol. (2023) 60:352–9. 10.1177/03009858231158075 36869834

[90] Gnat S Lagowski D Nowakiewicz A Dylag M A. global view on fungal infections in humans and animals: infections caused by dimorphic fungi and dermatophytoses. J Appl Microbiol. (2021) 131:2688–704. 10.1111/jam.15084 33754409

[91] Ashraf N Kubat RC Poplin V Adenis AA Denning DW Wright L. Re-drawing the maps for endemic mycoses. Mycopathologia. (2020) 185:843–65. 10.1007/s11046-020-00431-2 32040709 PMC7416457

[92] Olander HJ Reed H Pier AC. Feline cryptococcosis. J Am Vet Med Assoc. (1963) 142:138–43. 13939907

[93] Gionfriddo JR. Feline systemic fungal infections. Vet Clinics North Am Small Animal Pract. (2000) 30:1029–50. 10.1016/S0195-5616(00)05005-1 11033873

[94] Kurtz HJ Finco DR. Granulomatous chorioretinitis caused by Cryptococcus neoformans in a dog. J Am Vet Med Assoc. (1970) 157:934–7. 5528651

[95] Gelatt KN McGill LD Perman V. Ocular and systemic cryptococcosis in a dog. J Am Vet Med Assoc. (1973) 162:370–5. 4691372

[96] Angell JA Merideth RE Shively JN Sigler RL. Ocular lesions associated with coccidioidomycosis in dogs: 35 cases (1980-1985). J Am Vet Med Assoc. (1987) 190:1319–22. 3583890

[97] Greene RT Troy GC. Coccidioidomycosis in 48 cats: a retrospective study (1984-1993). J Vet Intern Med. (1995) 9:86–91. 10.1111/j.1939-1676.1995.tb03277.x 7760314

[98] Bergstrom BE Stiles J Townsend WM. Canine panuveitis: a retrospective evaluation of 55 cases (2000-2015). Vet Ophthalmol. (2017) 20:390–7. 10.1111/vop.12437 27734587

[99] Bloom JD Hamor RE Gerding PAJr. Ocular blastomycosis in dogs: 73 cases, 108 eyes (1985-1993). J Am Vet Med Assoc. (1996) 209:1271–4. 10.2460/javma.1996.209.07.1271 8837649

[100] Bromel C Sykes JE. Epidemiology, diagnosis, and treatment of blastomycosis in dogs and cats. Clin Tech Small Anim Pract. (2005) 20:233–9. 10.1053/j.ctsap.2005.07.004 16317913

[101] Buyukmihci N. Ocular lesions of blastomycosis in the dog. J Am Vet Med Assoc. (1982) 180:426–31. 7061329

[102] Morris JM Sigmund AB Ward DA Hendrix DVH. Ocular findings in cats with blastomycosis: 19 cases (1978-2019). J Am Vet Med Assoc. (2021) 260:422–7. 10.2460/javma.21.03.0135 34936573

[103] Pucket JD Fentiman KE McCool ES Hanzlicek AS. Prevalence of ocular lesions in cats newly diagnosed with histoplasmosis: 55 cases (2015-2022). J Am Vet Med Assoc. (2022) 260:1330–33. 10.2460/javma.22.03.0142 35594204

[104] Wilson AG KuKanich KS Hanzlicek AS Payton ME. Clinical signs, treatment, and prognostic factors for dogs with histoplasmosis. J Am Vet Med Assoc. (2018) 252:201–9. 10.2460/javma.252.2.201 29319442

[105] Simpson KW Khan KN Podell M Johnson SE Wilkie DA. Systemic mycosis caused by Acremonium sp in a dog. J Am Vet Med Assoc. (1993) 203:1296–9. 10.2460/javma.1993.203.09.1296 8253622

[106] Wooff PJ Dees DD Teixeria L. Aspergillus spp. panophthalmitis with intralenticular invasion in dogs: report of two cases. Vet Ophthalmol. (2018) 21:182–7. 10.1111/vop.12435 27641998

[107] Lincoln SD Adcock JL. Disseminated geotrichosis in a dog. Pathol Vet. (1968) 5:282–9. 10.1177/030098586800500308 5749201

[108] Baszler T Chandler FW Bertoy RW Smith CW Whiteley HE. Disseminated pseudallescheriasis in a dog. Vet Pathol. (1988) 25:95–7. 10.1177/030098588802500117 3344575

[109] Enders A van der Woerdt A Donovan T. Endogenous mycotic endophthalmitis in a dog with candiduria and Evans syndrome. Vet Ophthalmol. (2017) 20:84–8. 10.1111/vop.12373 26938883

[110] Elad D. Infections caused by fungi of the Scedosporium/Pseudallescheria complex in veterinary species. Vet J. (2011) 187:33–41. 10.1016/j.tvjl.2010.05.028 20580291

[111] Elad D. Disseminated canine mold infections. Vet J. (2019) 243:82–90. 10.1016/j.tvjl.2018.11.016 30606445

[112] Gerding PA Morton LD Dye JA. Ocular and disseminated candidiasis in an immunosuppressed cat. J Am Vet Med Assoc. (1994) 204:1635–8. 10.2460/javma.1994.204.10.1635 8050944

[113] Migaki G Font RL Sauer RM Kaplan W Miller RL. Canine protothecosis: review of the literature and report of an additional case. J Am Vet Med Assoc. (1982) 181:794–7. 6754671

[114] Stenner VJ Mackay B King T Barrs VR Irwin P Abraham L. Protothecosis in 17 Australian dogs and a review of the canine literature. Med Mycol. (2007) 45:249–66. 10.1080/13693780601187158 17464846

[115] Shank AM Dubielzig RD Teixeira LB. Canine ocular protothecosis: a review of 14 cases. Vet Ophthalmol. (2015) 18:437–42. 10.1111/vop.12239 25515728

[116] Pressler BM Gookin JL Sykes JE Wolf AM Vaden SL. Urinary tract manifestations of protothecosis in dogs. J Vet Inter Med. (2005) 19:115–9. 10.1111/j.1939-1676.2005.tb02669.x 15715059

[117] Masuda M Jagielski T Danesi P Falcaro C Bertola M Krockenberger M. Protothecosis in dogs and cats-new research directions. Mycopathologia. (2021) 186:143–52. 10.1007/s11046-020-00508-y 33206310

[118] Leiva M Naranjo C Pena MT. Ocular signs of canine monocytic ehrlichiosis: a retrospective study in dogs from Barcelona, Spain. Vet Ophthalmol. (2005) 8:387–93. 10.1111/j.1463-5224.2005.00409.x 16359361

[119] Stiles J. Canine rickettsial infections. Vet Clini North Am (Small Animal Practice). (2000) 30:1135–49. 10.1016/S0195-5616(00)05011-7 11033879

[120] Davidson MG Breitschwerdt EB Walker DH Levy MG Carlson CS Hardie EM. Vascular permeability and coagulation during Rickettsia rickettsii infection in dogs. Am J Vet Res. (1990) 51:165–70. 2105679

[121] Rutgers C Kowalski J Cole CR Sherding RG Chew DJ Davenport D. Severe Rocky Mountain spotted fever in five dogs. J Am Anim Hosp Assoc. (1985) 21:361–9.

[122] Michau TM Breitschwerdt EB Gilger BC Davidson MG. Bartonella vinsonii subspecies berkhoffi as a possible cause of anterior uveitis and choroiditis in a dog. Vet Ophthalmol. (2003) 6:299–304. 10.1111/j.1463-5224.2003.00310.x 14641826

[123] Dziezyc J. Canine systemic bacterial infections. Vet Clini North Am (Small Animal Practice). (2000) 30:1103–17. 10.1016/S0195-5616(00)05009-9 11033877

[124] Neer TM Breitschwerdt EB Greene RT Lappin MR. Consensus statement on ehrlichial disease of small animals from the infectious disease study group of the ACVIM. Am College Vet Int Med J Vet Int Med. (2002) 16:309–15. 10.1111/j.1939-1676.2002.tb02374.x 12041661

[125] Breitschwerdt EB Hegarty BC Hancock SI. Sequential evaluation of dogs naturally infected with Ehrlichia canis, Ehrlichia chaffeensis, Ehrlichia equi, Ehrlichia ewingii, or Bartonella vinsonii. J Clin Microbiol. (1998) 36:2645–51. 10.1128/JCM.36.9.2645-2651.1998 9705408 PMC105178

[126] Littman MP Gerber B Goldstein RE Labato MA Lappin MR Moore GE. consensus update on Lyme borreliosis in dogs and cats. J Vet Intern Med. (2018) 32:887–903. 10.1111/jvim.15085 29566442 PMC5980284

[127] Cohn LA. Ehrlichiosis and related infections. Vet Clinics North Am (Small Animal Practice). (2003) 33:863–84. 10.1016/S0195-5616(03)00031-7 12910747

[128] Breitschwerdt EB Davidson MG Hegarty BC Papich MG Grindem CB. Prednisolone at anti-inflammatory or immunosuppressive dosages in conjunction with doxycycline does not potentiate the severity of Rickettsia rickettsii infection in dogs. Antimicrob Agents Chemother. (1997) 41:141–7. 10.1128/AAC.41.1.141 8980770 PMC163675

[129] Abstracts: Abstracts: The 52nd annual scientific meeting of the American College of Veterinary Ophthalmologists Indianapolis IN Sept 29-Oct 2 2021. Vet Ophthalmol. (2021) 24:e1–e59. 10.1111/vop.12945 34708927

[130] Baneth G. Antiprotozoal treatment of canine babesiosis. Vet Parasitol. (2018) 254:58–63. 10.1016/j.vetpar.2018.03.001 29657012

[131] Meekins J Cino-Ozuna AG. Histologic identification of intraocular Cytauxzoon felis in three cats. JFMS Open Rep. (2018) 4:2055116918813242. 10.1177/2055116918813242 30559968 PMC6293365

[132] Gunn-Moore DA McFarland SE Schock A Brewer JI Crawshaw TR Clifton-Hadley RS. Mycobacterial disease in a population of 339 cats in Great Britain: II. Histopathology of 225 cases, and treatment and outcome of 184 cases. J Feline Med Surg. (2011) 13:945–52. 10.1016/j.jfms.2011.09.009 22061264 PMC10832975

[133] Stavinohova R O'Halloran C Newton JR Oliver JAC Scurrell E Gunn-Moore DA. Feline ocular mycobacteriosis: clinical presentation, histopathological features, and outcome. Vet Pathol. (2019) 56:749–60. 10.1177/0300985819844819 31132943

[134] Breitschwerdt EB Blann KR Stebbins ME Munana KR Davidson MG Jackson HA. Clinicopathological abnormalities and treatment response in 24 dogs seroreactive to Bartonella vinsonii (berkhoffii) antigens. J Am Anim Hosp Assoc. (2004) 40:92–101. 10.5326/0400092 15007043

[135] Lappin MR Black JC. Bartonella spp infection as a possible cause of uveitis in a cat. J Am Vet Med Assoc (1999). (1200) 214:1205–7. 10.2460/javma.1999.214.08.1205 10212684

[136] Ledbetter EC Landry MP Stokol T Kern TJ Messick JB. Brucella canis endophthalmitis in 3 dogs: clinical features, diagnosis, and treatment. Vet Ophthalmol. (2009) 12:183–91. 10.1111/j.1463-5224.2009.00690.x 19392878

[137] Andrew SE. Feline infectious peritonitis. Vet Clini North Am Small Animal Pract. (2000) 30:987–1000. 10.1016/S0195-5616(00)05002-6 PMC7134424 11033870

[138] Ziolkowska N Pazdzior-Czapula K Lewczuk B Mikulska-Skupien E Przybylska-Gornowicz B Kwiecinska K. Feline infectious peritonitis: immunohistochemical features of ocular inflammation and the distribution of viral antigens in structures of the eye. Vet Pathol. (2017) 54:933–44. 10.1177/0300985817728557 29065819

[139] Dickinson PJ Bannasch M Thomasy SM Murthy VD Vernau KM Liepnieks M. Antiviral treatment using the adenosine nucleoside analogue GS-441524 in cats with clinically diagnosed neurological feline infectious peritonitis. J Vet Intern Med. (2020) 34:1587–93. 10.1111/jvim.15780 32441826 PMC7379040

[140] Ledbetter EC Kim SG Dubovi EJ. Outbreak of ocular disease associated with naturally-acquired canine herpesvirus-1 infection in a closed domestic dog colony. Vet Ophthalmol. (2009) 12:242–7. 10.1111/j.1463-5224.2009.00709.x 19604340

[141] Albert DM Lahav M Carmichael LE Percy DH. Canine herpes-induced retinal dysplasia and associated ocular anomalies. Invest Ophthalmol. (1976) 15:267–78. 177383

[142] Fischer CA. Retinal and retinochoroidal lesions in early neuropathic canine distemper. J Am Vet Med Assoc. (1971) 158:740–52. 5103062

[143] Bistner SI. Ocular manifestations of systemic disease. Vet Clin North Am. (1973) 3:467–90. 10.1016/S0091-0279(73)50062-5 4365217

[144] Piper RC Cole CR Shadduck JA. Natural and experimental ocular toxoplasmosis in animals. Am J Ophthalmol. (1970) 69:662–8. 10.1016/0002-9394(70)91636-3 5437828

[145] Dubey JP Lindsay DS Lappin MR. Toxoplasmosis and other intestinal coccidial infections in cats and dogs. Vet Clinics North Am (Small Animal Practice). (2009) 39:1009–34. 10.1016/j.cvsm.2009.08.001 19932360

[146] Dubey JP Vianna MC Kwok OC Hill DE Miska KB Tuo W. Neosporosis in Beagle dogs: clinical signs, diagnosis, treatment, isolation and genetic characterization of Neospora caninum. Vet Parasitol. (2007) 149:158–66. 10.1016/j.vetpar.2007.08.013 17890012

[147] Pena MT Naranjo C Klauss G Fondevila D Leiva M Roura X. Histopathological features of ocular leishmaniosis in the dog. J Comp Pathol. (2008) 138:32–9. 10.1016/j.jcpa.2007.09.004 18048051

[148] Travi BL Cordeiro-da-Silva A Dantas-Torres F Miro G. Canine visceral leishmaniasis: diagnosis and management of the reservoir living among us. PLoS Negl Trop Dis. (2018) 12:e0006082. 10.1371/journal.pntd.0006082 29324838 PMC5764232

[149] Gwin RM Merideth RE Martin CL Kaswan RL. Ophthalmomyiasis interna posterior in two cats and a dog. J Am Anim Hosp Assoc. (1984) 30:481–6.

[150] Wyman M Starkey R Weisbrode S Filko D Grandstaff R Ferrebee E. Ophthalmomyiasis (interna posterior) of the posterior segment and central nervous system myiasis: Cuterebra spp. in a cat. Vet Ophthalmol. (2005) 8:77–80. 10.1111/j.1463-5224.2005.00343.x 15762919

[151] Edelmann ML Lucio-Forster A Kern TJ Bowman DD Ledbetter EC. Ophthalmomyiasis interna anterior in a dog: keratotomy and extraction of a Cuterebra sp. larva. Vet Ophthalmol. (2014) 17:448–53. 10.1111/vop.12210 25186977

[152] Ollivier FJ Barrie KP Mames RN Kallberg ME Greiner EC Plummer CE. Pars plana vitrectomy for the treatment of ophthalmomyiasis interna posterior in a dog. Vet Ophthalmol. (2006) 9:259–64. 10.1111/j.1463-5224.2006.00477.x 16771763

[153] Hughes PL Dubielzig RR Kazacos KR. Multifocal retinitis in New Zealand sheep dogs. Vet Pathol. (1987) 24:22–7. 10.1177/030098588702400105 3824819

[154] Perry AW Hertling R Kennedy MJ. Angiostrongylosis with disseminated larval infection associated with signs of ocular and nervous disease in an imported dog. Can Vet J. (1991) 32:430–1. 17423821 PMC1480994

[155] Aguirre GD Kazacos KR. Is it canine DUSN?: Another view of retinopathies, some acquired, and others possibly “inherited”: Another view of retinopathies, some acquired, and others possibly “inherited”. Vet Ophthalmol. (2022) 25:96–108. 10.1111/vop.12951 34894198 PMC10566749

[156] Johnson BW Kirkpatrick CE Whiteley HE Morton D Helper LC. Retinitis and intraocular larval migration in a group of Border Collies. J Am Anim Hosp Assoc. (1989) 25:623–9.

[157] Harcourt-Brown FM Holloway HK. Encephalitozoon cuniculi in pet rabbits. Vet Rec. (2003) 152:427–31. 10.1136/vr.152.14.427 12708591

[158] Magalhaes TR Pinto FF Queiroga FL. A multidisciplinary review about encephalitozoon cuniculi in a One Health perspective. Parasitol Res. (2022) 121:2463–79. 10.1007/s00436-022-07562-z 35840730 PMC9286959

[159] Nell B Csokai J Fuchs-Baumgartinger A Maass G. Encephalitozoon cuniculi causes focal anterior cataract and uveitis in dogs. Tierarztliche Praxis Ausgabe K, Kleintiere/Heimtiere. (2015) 43:337–44. 10.15654/TPK-141053 26355191

[160] Cray C Rivas Y. Seroprevalence of Encephalitozoon cuniculi in dogs in the United States. J Parasitol. (2013) 99:153–4. 10.1645/GE-3152.1 22694657

[161] T.S. de Boer Diaz Espineira MM Mandigers PJJ. Is encephalitozoon cuniculi of significance in young dogs with neurological signs? Front Vet Sci. (2021) 8:678968. 10.3389/fvets.2021.678968 34055959 PMC8149585

[162] Zarfoss MK Tusler CA Kass PH Montgomery K Lim CC Mowat F. Clinical findings and outcomes for dogs with uveodermatologic syndrome. J Am Vet Med Assoc. (2018) 252:1263–71. 10.2460/javma.252.10.1263 29701516 PMC13508167

[163] Cho H Jeong M Lee S Yoo S. Comparison of the qualitative and quantitative optical coherence tomographic features between sudden acquired retinal degeneration syndrome and normal eyes in dogs. Vet Ophthalmol 25 Suppl. (2022) 1:144–63. 10.1111/vop.12975 35144323

[164] Komaromy AM Abrams KL Heckenlively JR Lundy SK Maggs DJ Leeth CM. Sudden acquired retinal degeneration syndrome (SARDS) - a review and proposed strategies toward a better understanding of pathogenesis, early diagnosis, and therapy. Vet Ophthalmol. (2016) 19:319–31. 10.1111/vop.12291 26096588

[165] Carter RT Oliver JW Stepien RL Bentley E. Elevations in sex hormones in dogs with sudden acquired retinal degeneration syndrome (SARDS). J Am Anim Hosp Assoc. (2009) 45:207–14. 10.5326/0450207 19723843

[166] Oh A Foster ML Lunn KF Mowat FM. Circulating neurohormone imbalances in canine sudden acquired retinal degeneration syndrome and canine pituitary-dependent hypercortisolism. J Vet Intern Med. (2019) 33:2587–94. 10.1111/jvim.15646 31660652 PMC6872621

[167] Gelatt KN van der Woerdt A Ketring KL Andrew SE Brooks DE Biros DJ. Enrofloxacin-associated retinal degeneration in cats. Vet Ophthalmol. (2001) 4:99–106. 10.1046/j.1463-5224.2001.00182.x 11422990

[168] Sykes JE Blondeau JM. Pradofloxacin: a novel veterinary fluoroquinolone for treatment of bacterial infections in cats. Vet J.. (2014) 201:207–14. 10.1016/j.tvjl.2014.06.008 24997792

[169] Messias A Gekeler F Wegener A Dietz K Kohler K Zrenner E. Retinal safety of a new fluoroquinolone, pradofloxacin, in cats: assessment with electroretinography. Documenta ophthalmologica. Adv Ophthalmol. (2008) 116:177–91. 10.1007/s10633-007-9081-x 17909874

[170] Wiebe V Hamilton P. Fluoroquinolone-induced retinal degeneration in cats. J Am Vet Med Assoc. (2002) 221:1568–71. 10.2460/javma.2002.221.1568 12479325

[171] Meekins JM Guess SC Rankin AJ. Retinopathy associated with ivermectin toxicosis in five cats. J Am Vet Med Assoc. (2015) 246:1238–41. 10.2460/javma.246.11.1238 25970221

[172] Swor TM Whittenburg JL Chaffin MK. Ivermectin toxicosis in three adult horses. J Am Vet Med Assoc. (2009) 235:558–62. 10.2460/javma.235.5.558 19719447

[173] Pollio D Michau TM Weaver E Kuebelbeck KL. Electroretinographic changes after intravenous lipid emulsion therapy in a dog and a foal with ivermectin toxicosis. Vet Ophthalmol. (2018) 21:82–7. 10.1111/vop.12410 27440451

[174] Kenny PJ Vernau KM Puschner B Maggs DJ. Retinopathy associated with ivermectin toxicosis in two dogs. J Am Vet Med Assoc. (2008) 233:279–84. 10.2460/javma.233.2.279 18627233

[175] Merola VM Eubig PA. Toxicology of Avermectins and Milbemycins (Macrocyclic Lactones) and the Role of P-Glycoprotein in Dogs and Cats. Vet Clini North Am Small Animal Pract. (2018) 48:991–1012. 10.1016/j.cvsm.2018.07.002 30139545

[176] Epstein SE Hollingsworth SR. Ivermectin-induced blindness treated with intravenous lipid therapy in a dog. J Vet Emerg Crit Care (San Antonio). (2013) 23:58–62. 10.1111/vec.12016 23317101

[177] Chan RX Ledbetter EC. Sports ball projectile ocular trauma in dogs. Vet Ophthalmol. (2022) 25:338–42. 10.1111/vop.12987 35384230

[178] Leblanc NL Stepien RL Bentley E. Ocular lesions associated with systemic hypertension in dogs: 65 cases (2005-2007). J Am Vet Med Assoc. (2011) 238:915–21. 10.2460/javma.238.7.915 21453181 PMC4187359

[179] Maggio F DeFrancesco TC Atkins CE Pizzirani S Gilger BC Davidson MG. Ocular lesions associated with systemic hypertension in cats: 69 cases (1985-1998). J Am Vet Med Assoc. (2000) 217:695–702. 10.2460/javma.2000.217.695 10976302

[180] Young WM Zheng C Davidson MG Westermeyer HD. Visual outcome in cats with hypertensive chorioretinopathy. Vet Ophthalmol. (2019) 22:161–7. 10.1111/vop.12575 29667738

[181] Violette NP Ledbetter EC. Punctate retinal hemorrhage and its relation to ocular and systemic disease in dogs: 83 cases. Vet Ophthalmol. (2018) 21:233–9. 10.1111/vop.12496 28799185

[182] Gray HE Weigand CM Cottrill NB Willis AM Morgan RV. Polycythemia vera in a dog presenting with uveitis. J Am Anim Hosp Assoc. (2003) 39:355–60. 10.5326/0390355 12873025

[183] Tennant B Asbury AC Laben RC Richards WP Kaneko JJ Cupps PT. Familial polycythemia in cattle. J Am Vet Med Assoc. (1967) 150:1493–509. 6067913

[184] Harrus S Ofri R Aizenberg I Waner T. Acute blindness associated with monoclonal gammopathy induced by Ehrlichia canis infection. Vet Parasitol. (1998) 78:155–60. 10.1016/S0304-4017(98)00132-0 9735920

[185] Ramaiah SK Seguin MA Carwile HF Raskin RE. Biclonal gammopathy associated with immunoglobulin A in a dog with multiple myeloma. Vet Clini Pathol. (2002) 31:83–9. 10.1111/j.1939-165X.2002.tb00285.x 12040490

[186] Shelah-Goraly M Aroch I Kass PH Bruchim Y Ofri R A. prospective study of the association of anemia and thrombocytopenia with ocular lesions in dogs. Vet J.. (2009) 182:187–92. 10.1016/j.tvjl.2008.05.027 18664411

[187] Fischer CA. Retinopathy in anemic cats. J Am Vet Med Assoc. (1970) 156:1415–27. 5463038

[188] Brightman AH 2nd Ogilvie GK Tompkins M. Ocular disease in FeLV-positive cats: 11 cases (1981-1986). J Am Vet Med Assoc. (1991) 198:1049–51. 10.2460/javma.1991.198.06.1049 1851738

